# SMAD4 Palmitoylation Drives a Metabolic‐Transcriptional Circuit to Promote Tumorigenesis and Confers Radiosensitivity in Pancreatic Cancer

**DOI:** 10.1002/advs.202519791

**Published:** 2026-03-26

**Authors:** Yang Wang, Shan Zhang, Junping Bai, Xiaobing Li, Yi Han, Min Ji, Chongyi Jiang, Xiaobei Ge, Tianyu Yu, Yongying Hou, Jun Zhang, Wangting Li, Yiran Song, Jiaying Cai, Yingqun Zhou, Liwei An, Feng Wang

**Affiliations:** ^1^ Department of Gastroenterology Shanghai Tenth People's Hospital Tongji University School of Medicine Shanghai China; ^2^ YangPu District Mental Health Center Shanghai University of Medicine & Health Sciences Shanghai China; ^3^ Department of Stomatology Shanghai Tenth People's Hospital Tongji University Cancer Center Shanghai China; ^4^ Center For Molecular Recognition and Biosensing School of Life Sciences Shanghai University Shanghai China; ^5^ Department of Hepato‐Biliary‐Pancreatic Surgery Huadong Hospital Fudan University Shanghai China; ^6^ Department of Nursing Huadong Hospital Fudan University Shanghai China; ^7^ Hepatobiliary Surgery Center Shanghai Tongji Hospital Affiliated to Tongji University Tongji University Shanghai China; ^8^ Department of Pathology Daping Hospital Army Medical University (Third Military Medical University) Chongqing China; ^9^ International Cancer Center Guangdong Key Laboratory of Genome Instability and Human Disease Prevention Department of Biochemistry and Molecular Biology Shenzhen University Medical School Shenzhen China; ^10^ School of Basic Medicine and Clinical Pharmacy China Pharmaceutical University Nanjing China; ^11^ Department of Gastroenterology Shanghai General Hospital Shanghai JiaoTong University School of Medicine Shanghai China; ^12^ Department of Gastroenterology Huadong Hospital Shanghai Medical College Fudan University Shanghai China

**Keywords:** FASN, palmitic acid, palmitoylation, pancreatic cancer, radiotherapy, SMAD4

## Abstract

Pancreatic ductal adenocarcinoma (PDAC) is characterized by frequent SMAD4 inactivation and profound lipid metabolic rewiring, yet how these processes intersect especially in SMAD4^+^ PDAC remains elusive. Here, we identify palmitoylation as a previously unrecognized post‐translational modification of SMAD4. Combining biochemical labeling, mutagenesis, and functional assays, we demonstrate that SMAD4 is palmitoylated at cysteine 363 by the acyltransferase ZDHHC22 and depalmitoylated by APT2. Mechanistically, palmitoylation enhances SMAD4 protein stability, facilitates the interaction of SMAD4 with importins, and amplifies subsequent transcriptional output, leading to direct upregulation of the key fatty acid biosynthetic enzyme FASN. Consequently, elevated palmitic acid levels in turn reinforce SMAD4 palmitoylation, establishing a self‐amplifying SMAD4 palmitoylation–FASN–palmitic acid positive feedback loop that drives PDAC tumor growth. Intriguingly, SMAD4 palmitoylation sensitizes PDAC cells to radiotherapy both in vitro and in vivo, revealing a dual role between tumor progression and treatment responses. Notably, clinically relevant SMAD4 mutants (R361C and R361H) exhibit enhanced palmitoylation, underscoring the pathological relevance of this mechanism for tumorigenesis. Collectively, these findings unveil a metabolic‐transcriptional circuit wherein palmitoylation bridges lipid metabolism with SMAD4‐driven oncogenesis, and posit SMAD4 palmitoylation as a therapeutic vulnerability in pancreatic cancer.

## Graphical abstract

This study identifies palmitoylation as a novel regulatory modification of SMAD4, mediated by ZDHHC22/APT2. It activates fatty acid synthesis, creating a self‐reinforcing SMAD4–FASN–palmitate feedback loop that drives pancreatic cancer growth and enhances radiotherapy sensitivity. This metabolic‐transcriptional circuit represents a promising therapeutic target for PDAC patients.

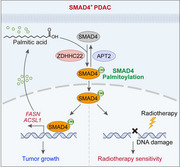

## 1 Introduction

Pancreatic ductal adenocarcinoma (PDAC) is one of the most aggressive malignancies and ranks as the sixth leading cause of cancer‐related deaths globally among both men and women [1]. It's extremely poor prognosis is largely attributable to the various factors, including its low early‐detection rate, rapid progression and resistance to chemotherapy [2, 3]. Genomic profiling has revealed frequent somatic gene mutations including the oncogene *KRAS*, along with tumor suppressors *CDKN2A*, *TP53* and *SMAD4*, are observed in PDAC. Notably, *SMAD4* mutations, present in approximately 50% of PDAC cases, are strongly associated with radiotherapy resistance and significantly poorer prognosis outcomes [4, 5, 6, 7]. Nevertheless, in the remaining 40%–50% PDAC cases with intact SMAD4 expression, the role of SMAD4 in tumorigenesis remains poorly defined. This gap highlights the pressing need to elucidate the regulatory mechanisms governing SMAD4 in PDAC and to develop targeted therapeutic strategies that address SMAD4‐related vulnerabilities.

In parallel with genetic drivers, PDAC is profoundly influenced by metabolic reprogramming, particularly in lipid metabolism, which supports rapid proliferation and adaptation to nutrient stress [8, 9]. Palmitic acid (PA), the most abundant saturated fatty acid in the human body, constitutes 20%–30% of the total fatty acids and can be acquired through dietary intake or endogenous lipogenesis [10]. Beyond serving as a structural lipid, PA functions as a potent bioactive metabolite in cancer biology. For example, dietary PA has been reported to promote tumor metastasis [11]. and a PA‐driven ZDHHC15 YAP palmitoylation feedback loop was recently identified to accelerate metastatic processes [12]. These studies highlight that PA can act not only as a metabolic substrate but also as a signaling molecule that promotes oncogenesis.

One of the primary mechanisms by which PA exerts its regulatory role is protein S‐palmitoylation, a reversible post‐translational modification, in which palmitate is covalently linked to cysteine residues via thioester bonds, is estimated to affect 10%–20% of the human proteome [13]. Palmitoylation critically modulates protein localization, stability, and protein–protein interactions, thereby impacting diverse cell signaling pathways and biological processes [14]. For instance, the palmitoylation of GPX4, enhances its protein stability and governs ferroptosis sensitivity and antitumor immunity [15, 16]. In PDAC, ZDHHC20‐mediated S‐palmitoylation of the m6A reader YTHDF3 has been reported to stabilize MYC mRNA, promoting the progression of KRAS‐mutant pancreatic cancer [17]. Despite these advances, the scope and mechanistic contribution of additional protein palmitoylation events in PDAC remains to be elucidated.

The activation status of SMAD family proteins is intricately regulated by a diverse repertoire of post‐translational modifications (PTMs), such as (de)phosphorylation [18], (de)ubiquitylation [19, 20], sumoylation [21, 22, 23], (de)‐acetylation, ADP‐ribosylation and methylation [23, 24, 25, 26]. Each PTM introduces a distinct layer of regulation, enabling SMAD signaling to dynamically respond to varying cellular environments and external stimuli. As a central transcription factor within the TGF‐β pathway, SMAD4 plays an indispensable role in signal integration and transcriptional output [27]. Upon TGF‐β pathway activation, SMAD2 and SMAD3 associate with SMAD4 to form a heterotrimeric complex, which translocates into the nucleus to orchestrate target gene expression. For example, SMAD4 sumoylation has been shown to modulate its transcriptional activity [21], while methylation by PRMT5 promotes oncogenic signaling in colorectal cancer [24]. Despite this growing knowledge, the full spectrum of SMAD4‐specific PTMs, including palmitoylation, particularly in the context of PDAC, remains incompletely understood.

In this study, we identify palmitoylation as a novel regulatory mechanism of SMAD4 in PDAC. Our findings demonstrate that SMAD4 promotes FASN‐dependent palmitic acid synthesis and that SMAD4 itself undergoes palmitoylation at cysteine 363. Functionally, palmitoylated SMAD4 facilitates gene transcriptional activity especially those related to fatty acid biosynthesis, such as FASN, thereby linking SMAD4 activity to lipid metabolic programs. Mechanistically, we identified that ZDHHC22 is the palmitoyl acyltransferase, while acyl protein thioesterase (APT)2 mediates the depalmitoylation of SMAD4, which in turn regulates SMAD4 protein stability and the interaction with nuclear importins. Functionally, we revealed a dual role of nuclear palmitoylated SMAD4 between tumor progression and treatment responses. Taken together, our findings reveal a SMAD4 palmitoylation‐FASN‐palmitic acid feedback loop that could serve as a potential therapeutic target for PDAC.

## 2 Materials and Methods

### 2.1 Cell Lines and Culture Conditions

The human pancreatic ductal adenocarcinoma cell lines Panc‐1 and Bxpc‐3, as well as the human embryonic kidney cell line HEK293FT, were purchased from the cell library of the Chinese Academy of Sciences (Shanghai, China). Panc‐1 and HEK293FT cells were cultured in Dulbecco's modified Eagle's medium (DMEM, Gibco, USA) supplemented with 10% fetal bovine serum (FBS, Gibco, USA) with 100 µg/mL penicillin and 100 µg/mL streptomycin. Bxpc‐3 cells were maintained in Roswell Park Memorial Institute (RPMI) 1640 medium (Gibco, USA) containing 10% FBS and 100 µg/mL of penicillin/streptomycin. All cells were continuously cultured at 37°C with 5% CO_2_, and subcultured using 0.25% trypsin‐EDTA (Gibco, USA) upon reaching 70%–80% confluence.

### 2.2 Plasmids

Full‐length human SMAD4 cDNA and its mutants, were subcloned into a modified pCDH lentiviral vector containing an N‐terminal 3×Flag tag. These constructs were also introduced into pLV‐EGFP tagged with a GFP tag and pLV vectors with an HA tag, respectively.

cDNAs encoding the 23 members of the ZDHHC family, as well as depalmitoylases APT1, APT2, PPT1, ABHD10, ABHD17A, ABHD17B, and ABHD17C, were amplified from human cDNA libraries and subcloned into the modified pCDH vector with an N‐terminal 3×Flag tag. All resulting plasmid constructs were verified by performing DNA sequencing (GENEWIZ Biotechnology, China) to ensure sequence accuracy.

### 2.3 Transfection, Lentivirus Packaging, and Infection

For transient transfection, plasmid constructs were pre‐incubated with polyethyleneimine (PEI, Cat# 23966‐1, Polysciences, USA) at a ratio of 1:4 in Opti‐MEM (Thermo Fisher Scientific, USA). The mixture was incubated at room temperature for 15 min before being added dropwise to the cell culture medium.

For the production of lentiviral particles, lentiviral‐based constructs were co‐transfected with the packaging plasmids psPAX2 and pMD2.G into HEK293FT cells in Opti‐MEM at a mass ratio of 4:3:1 using PEI. The resulting supernatants containing lentiviruses were collected at 24, 48, and 72 h after transfection, filtered through a 0.45 µm pore size filter, and stored at −80°C. Target cells were infected with lentiviruses in the presence of 10 µg/mL polybrene (Cat# 28728‐55‐4, Sigma‐Aldrich, USA). Additionally, for SMAD4 knockdown, lentiviral vectors carrying human SMAD4‐specific shRNA with the target sequence as follows: shSMAD4‐1: 5’‐GGTGTGCAGTTGGAATGTA‐3’; shSMAD4‐2: 5’‐GATTAACACTGCAGAGTAA‐3’, along with non‐targeting controls, were purchased from Genechem (China). Infection and selection were performed as described above, with knockdown efficiency verified by western blotting.

### 2.4 Small Interfering RNA (siRNA) Transfection

Cells were grown to confluence on 6‐cm tissue culture plates, and transfected with one of the 4 APT2‐specific siRNA oligos (si‐APT2‐a: 5’‐CCUACACCAUGUCUCGAAUTT‐3’; si‐APT2‐b: 5’‐CUGGUCGUGUGAUUGACAUTT‐3’; si‐APT2‐c: 5’‐CUCCUUUGCAUAUCCCAAUTT‐3’; si‐APT2‐d: 5’‐GGAGCAUGGAAUAUACACATT‐3’) and ZDHHC22‐specific siRNA oligos (si‐ZDHHC22: 5’‐GGTTCATTTATGCCCTATA‐3’) [28] using GP‐transfect‐Mate (GenePharma, China) as the transfection reagent according to the manufacturer's instruction. A scrambled siRNA (siScr: 5’‐UUCUCCGAACGUGUCACGUTT ‐3’) was applied as a negative control. Cells were harvested 72 h later for gene expression analysis by western blot and qPCR.

### 2.5 Chemical Agents

Chemical agents used included the radiomimetic drug NCS (Cat# 9014‐02‐2, Sigma‐Aldrich, USA), Etoposide (Cat# S1225, Selleck, USA), ML349 (Cat# HY‐100737, MedChemExpress, USA) and 2‐BP (Cat# HY‐111770, MedChemExpress, USA).

### 2.6 Immunoprecipitation and Western Blot Analysis

For the co‐immunoprecipitation (co‐IP) assays, cells were lysed using NETN buffer (20 mM Tris‐HCl, pH 8.0, 100 mM NaCl, 1 mM EDTA, and 0.5% NP‐40) for 30 min at 4°C. Lysates were then centrifuged at 12 000 rpm for 15 min at 4°C. The resulting clarified supernatants were incubated overnight at 4°C with rotation using anti‐DYKDDDDK affinity beads (Cat# SA042025, Smart‐Lifesciences, China), anti‐GFP affinity beads (Cat# SA070025, Smart‐Lifesciences, China), streptavidin beads (Cat# SA021100, Smart‐Lifesciences, China), or Protein A/G PLUS Agarose beads (Cat# sc‐2003, Santa Cruz Biotechnology, USA) conjugated with the appropriate antibodies. Post‐incubation, beads were washed thrice with NETN buffer and eluted by boiling them in 1× sodium dodecyl sulfate polyacrylamide gel electrophoresis (SDS‐PAGE) loading buffer. To minimize interference from immunoglobulin light/heavy chains, specific horseradish peroxidase (HRP)‐conjugated secondary antibodies (Cat# M21008, Abmart, China) were used at a 1:500 dilution. The antibodies used in this study are listed as follows: anti‐Flag (Sigma, F3165, diluted at 1:2000), anti‐SMAD4 (Cell Signaling Technology, 46535, diluted at 1:1000), anti‐β‐Actin (Cell Signaling Technology, 4967, diluted at 1:2000), anti‐Streptavidin (Abcam, ab7403, diluted at 1:1000), anti‐FASN (MedChemExpress, HY‐P80668, diluted at 1:1000), anti‐HA (Cell Signaling Technology, 3724, diluted at 1:2000), anti‐GFP (Santa Cruz Biotechnology, sc‐9996, diluted at 1:2000), anti‐Histon H3 (Abmart, T56587, diluted at 1:4000), anti‐ GAPDH (Abmart, P60037, diluted at 1:4000), anti‐Importin 7 (Abcam, ab99273, diluted at 1:1000) and anti‐Importin 8 (Abmart, A14679, diluted at 1:1000) are used for WB.

### 2.7 Immunofluorescence Staining

To carry out immunofluorescence staining, the target cells were seeded onto glass‐bottom dishes (Cat# D29‐20‐1.5‐N, Cellvis, USA), and after 24 h washed thrice with phosphate‐buffered saline (PBS) and fixed with 1 mL of 4% paraformaldehyde at room temperature for 10 min. Then they were permeabilized with 1 mL of 0.5% Triton X‐100 (Cat# A600198, Sangon Biotech, China) for 1 min. After three washes with PBS, the cells were incubated with 3% bovine serum albumin (BSA) for 30 min. Subsequently, the cells were incubated for 2 h at room temperature with primary antibodies, diluted in primary antibody dilution buffer (Cat# P0023A, Beyotime Biotechnology, China). After three washes with PBS, the cells were incubated for 1 h at room temperature in the dark with fluorochrome‐conjugated secondary antibodies diluted in the primary antibody dilution buffer. The resulting cells were subjected to three more washes with PBS, and then to nuclear staining using 0.2 µg/mL DAPI for 5 min in the dark, followed by a final wash with PBS. Images of the cells were acquired using a confocal microscope (ZEISS, Germany). Anti‐Flag (Sigma, F3165, diluted at 1:200), anti‐SMAD4 (Cell Signaling Technology, 46535, diluted at 1:200) and anti‐γ‐H2AX (Sigma, 05‐636, diluted at 1:200) are used for IF.

### 2.8 BODIPY Staining

Cells were seeded into glass‐bottomed dishes (Cat# D29‐20‐1.5‐N, Cellvis, USA). After 24 h the medium was removed, and cells were washed thrice with pre‐warmed phosphate‐buffered saline (PBS) to eliminate serum interfering with dye binding before staining. BODIPY 493/503 (Cat# HY‐W090090, MedChemExpress, USA) staining solution at 1.0 µg/mL was added and samples were incubated in a dark environment for 1 h. After incubation, the staining solution was removed, and cells were washed three times with PBS to remove unbound dye. Images of the cells were acquired using a confocal microscope (ZEISS, Germany).

### 2.9 Cell Viability Assay

Cells were seeded into 96‐well plates at a density of 1000 per well overnight. The cells were treated  for 48 h, and then used with an Cell Counting Kit‐8 (Cat#40203ES92, Yeasen, China) according to the manufacturer's instructions. The absorbance was measured at 450 nm wave length and all experiments were performed at least three times.

### 2.10 RNA Extraction and qRT‒PCR

Total RNA was extracted using a MolPure Cell/Tissue Total RNA Kit (Cat# 19221ES50, Yeasen, China), and then subjected to qRT‐PCR analysis.

For qRT‐PCR analysis, 1 µg of total RNA was used for cDNA synthesis from the total RNA using a Hifair III first Strand cDNA Synthesis SuperMix for qPCR kit (Cat#11141ES60, Yeasen, China) following the manufacturer's instructions. qPCRs were processed using 2× Universal SYBR Green Fast qPCR Mix (Cat# RM21203, ABclonal, China). qPCR primer sequences are listed as follows: *β‐Actin*‐F: AGCGAGCATCCCCCAAAGTT, *β‐Actin*‐R: GGGCACGAAGGCTCATCATT; *SMAD4*‐F: CCTGTGGCTTCCACAAGTC, *SMAD4*‐R: CTGATGCTATCTGCAACAGTCC; *FASN*‐F: CGCGTGGCCGGCTACTCCTAC; *FASN*‐R: CGGCTGCCACACGCTCCTCT; *ZDHHC22*‐F: GGGGCGCTCTTCCTATTCC, *ZDHHC22*‐R: GCAGAAGTGGGTGCTAGGTG [28]; *APT2*‐F: TGGACGCGGCTTTACTGTG, *APT2*‐R: GCAGCCCTCTTAGCTTTAGCA.

### 2.11 Immunohistochemical (IHC) Staining

For IHC staining, the tissues were embedded in paraffin and sectioned into 5 µm slices, then deparaffinized in xylene, rehydrated through a graded series of ethanol (100%, 95%, 80%, 70%; 5 min each), and rinsed three times in PBST for 5 min each. Subsequently, antigen retrieval was performed by boiling the sections in 10 mM citrate buffer (pH 6.0) using a 750 W microwave oven for 15 min, followed by natural cooling to room temperature. Non‐specific binding was blocked with 5% goat serum in PBST for 1 h. Primary antibodies used were anti‐SMAD4 (Abcam, ab40759, diluted at 1:100), anti‐FASN (Abcam, ab128870, diluted at 1:450), anti‐Ki‐67 (Cell Signaling Technology, 9449, diluted at 1:100), anti‐ZDHHC22 (Santa Cruz Biotechnology, sc‐514005, 1:50) and anti‐APT2 (Santa Cruz Biotechnology, sc‐390546, 1:50). After three 5 min rinses in PBST, sections were incubated with HRP‐conjugated goat anti‐rabbit or anti‐mouse IgG at room temperature for 1 h, developed with DAB substrate (ZSGB‐BIO) for 2–5 min (monitored under a light microscope to avoid over‐staining), and counterstained with hematoxylin for 30 s. Slides were dehydrated, cleared in xylene, and mounted with neutral balsam. Tissue microarray sections were constructed and provided by PowerX‐Bio (China).

The IHC staining was scored semi‐quantitatively by two independent pathologists in a double‐blinded manner. Staining intensity was graded as follows: 0 (negative), 1 (weak), 2 (moderate), and 3 (strong). The proportion of positive‐staining cancer cells was scored as: 1 (1%–25%), 2 (26%–50%), 3 (51%–75%), and 4 (76%–100%). The final IHC score was calculated as the product of intensity and positive cell proportion.

### 2.12 Free Fatty Acid (FFA) Analysis

Tissues were harvested, transferred to centrifuge tubes, homogenized and centrifuged at 12 000 g for 5 min, then the supernatant was discarded. Quantification of FFA content was conducted using an Amplex Red Free Fatty Acid Assay Kit (Cat#S0215M, Beyotime, China) in accordance with the provided guidelines.

### 2.13 Acyl‐Biotinyl Exchange (ABE) Assay

The ABE assay was performed using the IP‐ABE Palmitoylation Kit for western blots (Cat#AM103134, AIMSMASS, China) according to the manufacturer's instructions. Briefly, the procedure included blocking, reduction, labelling, elution and detection. Cells were harvested and suspended in lysis buffer, followed by incubation with beads and anti‐HA or anti‐Flag overnight at 4°C. N‐ethylmaleimide (NEM) was used to block the unmodified cysteines for 30 min. Then, the beads were washed and each group was divided into two parts, one incubated with hydroxylamine (HAM) for 1 h at room temperature (+ HAM) and one omitting the HAM cleavage step (‐HAM). After washing, the beads were treated with thiol‐reactive biotin molecules for 1 h at room temperature. The beads were boiled for 10 min after resuspending in SDS‐PAGE loading buffer and then subjected to western blotting analysis with Streptavidin‐HRP antibody.

### 2.14 Click Chemistry to Detect Palmitoylation

In this study, the click chemistry method was utilized to detect palmitoylation proteins. For LC‐MS/MS samples, Panc‐1 cells were incubated with alkynyl palmitic acid (50 µM) for 24 h. To detect SMAD4 palmitoylation, the plasmid was transfected into HEK293FT cells, which were harvested and lysed after 48 h, and then alkynyl palmitic acid (50 µM) was added to the lysate and incubated at 37°C for 2 h. Cells were collected and treated with pre‐cooled RIPA lysis buffer for 15 min on ice. Ten percent of each lysate was used as the assay input, while the remainder was used for labeling palmitoylation proteins.

Copper‐catalyzed click chemistry was used to conjugate alkynyl palmitic acid with the Azide‐Biotin molecule. Specifically, 100 µM Azide‐Biotin (Cat#CCT‐1265,Vectorlabs, USA) was added to the sample, followed by the addition of 100 µM Tris[(1‐benzyl‐1H‐1,2,3‐triazol‐4‐yl)methyl]amine (TBTA) (Cat#678937, Sigma, USA), 1 mM Tris(2‐carboxyethyl)phosphine hydrochloride (TCEP) (Cat#C4706, Sigma, USA), 1 mM CuSO [4] (Cat#C1297, Sigma, USA), and 2.5 mM sodium ascorbate (Cat#11140, Sigma, USA). The mixture was allowed to react at room temperature for 1 h. After completing the labeling reaction, the proteins were precipitated with pre‐cooled methanol and stored at −80°C overnight. Next, the samples were centrifuged at 8, 000 g for 5 min at 4°C, the supernatant was discarded, and the protein pellets were washed with pre‐cooled methanol. Subsequently, the protein pellets were resuspended in 0.5% SDS buffer. Streptavidin agarose beads were added to the samples and rotated at room temperature for 1 h. The beads were collected and washed with 8 M urea to remove non‐covalently bound proteins, followed by three washes with RIPA lysis buffer. The washed beads were suspended in 1×SDS loading buffer and heated at 99°C for 10 min.

### 2.15 Neutral Comet Assay

Cells were harvested for the neutral comet assay [29], which was performed using the Comet SCGE assay kit (Cat# ADI‐900‐166, Enzo, USA) in strict accordance with the manufacturer's instructions. Briefly, first cells were subjected to 10 Gy of IR and collected at 4 h post IR. After being rinsed twice with ice‐cold PBS, cells were harvested at about 1 × 10^5^ cells/ml and mixed with molten LMAgarose (4250‐050‐02, Enzo) at a ratio of 1:10 (v/v). The mixture was immediately pipetted onto a Comet Slide apparatus (4250‐050‐03, Enzo). Each slide with the mixture was put into a 4°C refrigerator for 30 min and then treated with neutral lysis buffer (2.5 M NaCl, 100 mM Na_2_EDTA, 1% sodium lauroyl sarcosine, 10 mM Tris, pH 7.5, 1% Triton‐X100 and 10% DMSO) overnight. Next, each treated slide was subjected to electrophoresis at 25 V for 30 min and stained in CYGREEN Nucleic Acid Dye agent for 30 min. Images were captured using a fluorescence microscope (Olympus BX51) and analyzed with CometScore software.

### 2.16 CUT&Tag Assay

Panc‐1 cells were harvested for the CUT‐Tag assay, which was performed using the Hyperactive Universal CUT&Tag Assay Kit for Illumina Pro (Cat# TD904, Vazyme Biotech, Nanjing, China) in strict accordance with the manufacturer's instructions. In brief, ConA beads were added in each sample for pre‐incubation at room temperature for 10 min and then incubated with the primary antibody, anti‐SMAD4 (Cell Signaling Technology, 46535), or IgG overnight at 4°C. The secondary antibody incubation was employed at room temperature for 1 h and pA/G‐Tnp pro incubation was employed at room temperature for 1 h. Fragmentation, treatment of DNA Extract Beads Pro, DNA Extraction, Library Amplification and PCR product purification were employed to get CUT&Tag fragments DNA libraries. The DNA libraries were amplified for 15 cycles and sequenced using Illumina NovaSeq X plus (APExBIO Technology LLC).

Raw sequencing reads were preprocessed to filter out sequencing adapters, short reads (length <30 bp), and other low‐quality reads. The resulting clean reads were aligned to the human reference genome (hg38) using Bowtie (v2.5.3). Peak calling was performed using the MACS2 (version 2.2.9.1) with default parameters except for adjusting the q‐value (FDR) cut‐off to 0.05 to determine significant peaks. Differentially peaks were identified by MAnorm (v1.3.0) with a cut‐off value of log2|fold‐change|≥1 and *p*‐value≤0.01. The clusterProfiler (v4.10.0) was employed to perform functional enrichment analysis for the annotated significant peaks based on Gene Ontology (GO) and the Kyoto Encyclopedia of Genes and Genomes (KEGG).

### 2.17 CUT&RUN Assay

Panc‐1 cells were collected for CUT&RUN assay using Hyperactive pG‐MNase CUT&RUN Assay Kit for PCR/Qpcr (Cat# HD101‐01, Vazyme, China). Panc‐1 cells were incubated with ConA Beads at room temperature for 10 min, and then incubated with the primary antibody, anti‐SMAD4 (Cell Signaling Technology, 46535) or IgG overnight at 4°C. The following day, they were incubated with pG‐MNase enzyme at 4°C for 1 h. Fragmentation, fragmentation termination and DNA fragment release, DNA extraction were employed to get the products for qPCR. Primer sequences are listed as follows: *FASN*‐promoter‐F: TTTTTGAGACAGAGTCTTGCTCT, *FASN*‐promoter‐R: CCAGCCTGGGCGACAG; *ACSL1*‐promoter‐F: AGCTGCGGGCGCTGCCCA, *ACSL1*‐promoter‐R: GCGCGGCCCCACAGGGTT; *ACACA*‐promoter‐F: ATTGGCTGCCGCTGAGCC, *ACACA*‐promoter‐R: ACGTCCGGGGTTACTCCAGG.

### 2.18 Spatial Metabolomics Analysis

To investigate spatial metabolic alterations, an orthotopic pancreatic cancer model was established by inoculating 1mm^3^ into the pancreas of male BALB/c nude mice. 2 weeks post‐inoculation, mice were sacrificed, and tumor tissues were rapidly harvested, rinsed with pre‐cooled PBS, and flash‐frozen in liquid nitrogen. Frozen tissues were sectioned at 10 µm thickness using a cryostat and thaw‐mounted on positive charge desorption plate (Thermo Scientific, U.S.A.). The samples were submitted to OE Biotech (Shanghai, China) for spatial metabolomics analysis, the experiment was carried out with a AFADESI‐MSI platform (Beijing Victor Technology Co., LTD, Beijing, China) in tandem with a Q‐Orbitrap mass spectrometer (Q Exactive, Thermo Scientific, U.S.A.). MSI data acquisition was performed on a DESI‐Orbitrap mass spectrometer (Thermo Fisher Scientific) in positive and negative ion modes with a spatial resolution of 100 µm and a mass range of m/z 70–1200. Meanwhile, adjacent sections were stained with hematoxylin and eosin (H&E) and evaluated by pathologists to annotate histological regions, including tumor parenchyma and normal tissue. Raw data were converted to the standard “imzML” format and imported into MSiReader software for visualization. Region‐specific metabolic profiles were extracted by co‐registering the MSI ion images with high‐resolution H&E‐stained images to ensure accurate spatial extraction. Multivariate statistical analysis was performed to identify discriminating metabolites between groups. Variable Importance in Projection (VIP) values were calculated using Orthogonal Partial Least Squares Discriminant Analysis (OPLS‐DA), and differential metabolites were defined based on a VIP value>1.0 and a statistically significant difference (*p* < 0.05) determined by a two‐tailed Student's *t*‐test.

### 2.19 RNA Sequencing

Total RNA was extracted using TRIzol reagent (Cat# R401‐01, Vazyme, China) and then submitted to OE Biotech (Shanghai, China) for RNA sequencing. About 7.15‐7.43 G raw reads produced per sample. Raw reads in FASTQ format were first processed using fastp (version 0.20.1) to remove low‐quality reads, resulting in 6.81–7.04 G clean reads retained per sample for subsequent analyses. Clean reads were aligned to reference genome using the Hisat2 software (version 2.1.0). The number of read counts per gene was quantified using HTSeq‐count (version 0.11.2), and the FPKM (Fragments Per Kilobase of transcript per Million mapped fragments) value of each gene was further calculated. Differential expression analysis was conducted using the DESeq2 (version 1.22.2), with genes defined as significantly differentially expressed genes (DEGs) based on the thresholds of adjusted *p*‐value (*q*‐value)<0.05 and fold change (FC)>2 or FC<0.5.

### 2.20 Untargeted Metabolomics

Serum samples of PDAC patients were harvested for the measurement of untargeted metabolomics performed by Majorbio (Majorbio Biotech Co., Ltd., Shanghai, China). In brief, 300 µL solution (methanol: water = 4:1 (v:v)) containing 0.02 mg/mL of internal standard (L‐2‐chlorophenylalanine) used for metabolite extraction was added to 100 µL sample. Then the samples were mixed by vortex for 30 s and subjected to low‐temperature sonicated for 30 min (5°C, 40 KHz). To facilitate protein precipitation, samples were placed at −20°C for 30 min. followed by centrifugation for 15 min (4°C, 13 000 g). The resulting supernatant was removed and blown dry under nitrogen. The residue was then re‐solubilized with 100 µL solution (acetonitrile: water = 1:1) and extracted by low‐temperature ultrasonication for 5 min (5°C, 40 KHz), followed by centrifugation for 10 min (4°C, 13 000 g). The final supernatant was transferred to sample vials for LC‐MS/MS analysis. The mass spectrometric data were collected using a Thermo UHPLC‐Q Exactive HF‐X Mass Spectrometer. The raw data were uploaded into Progenesis QI v3.0 (WatersCorporation, Milford, USA) for peak detection and alignment. The data analyses were performed on the free online platform of Majorbio cloud platform. The metabolites with VIP>1.0, adjusted *p*‐value (*q*‐value)<0.05, and fold change (FC)>1.2 or FC<0.83 were considered to be differential metabolites (DEMs).

### 2.21 Mass Spectrometry

For LC‐MS/MS samples, Panc‐1 cells or PDAC tumor tissues were incubated with alkynyl palmitic acid (50 µM) for 24 h. After click chemistry, the IP samples were subjected to mass spectrometric (MS) analysis by QLBio (Beijing, China).

### 2.22 Human Participants

Patients diagnosed with PDAC are enrolled in this study. All the procedures were approved by the ethical review board at Huadong Hospital (No. 2021K132). 20 healthy individuals and 20 PDAC patients were collected serum samples for untargeted metabolomics. 3 tumor tissues of PDAC patients were collected for click chemistry. 20 tumor tissues of PDAC patients were collected for free fatty acid assay. 17 tumor tissues of PDAC patients were collected for IHC analysis. Tumor tissues and normal tissues were taken from patients with PDAC during surgery under the patients’ informed consent.

### 2.23 In Vivo Mouse Model Assays

Male BALB/c nude mice, aged 3–4 weeks, were purchased from Shanghai Model Organisms Center (Shanghai, China). All mice were housed in a standard animal facility at 22°C, with access to sterile standard food and water. The care and use of the animals were approved by the ethical review board at Shanghai Tenth People's Hospital (No. SHDSYY‐2024‐2532). All procedures were performed in accordance with the relevant guidelines and regulations. For tumor induction, 5 × 10^6^ cells were injected subcutaneously into the right flank of each mouse. Tumor size was monitored every 3 days and tumor volume was estimated as 0.52 × length × width × width. After 4 weeks, the mice were sacrificed, and tumors were dissected and weighed.

### 2.24 Statistical Analysis

Data for statistical analysis were derived from at least three independent experiments and were analyzed with GraphPad Prism 8.0 statistical software. Student's *t*‐test was employed to assess statistical significance to compare two variables. Survival outcomes were analyzed using the log‐rank test. Values of *p* < 0.05 were considered to indicate statistical significance. Survival curves were calculated according to the Kaplan–Meier method; survival analysis was performed using the log‐rank test.

## 3 Results

### 3.1 SMAD4 Expression Correlates With Palmitic Acid Levels Within TME of PDAC Patients

**FIGURE 1:**
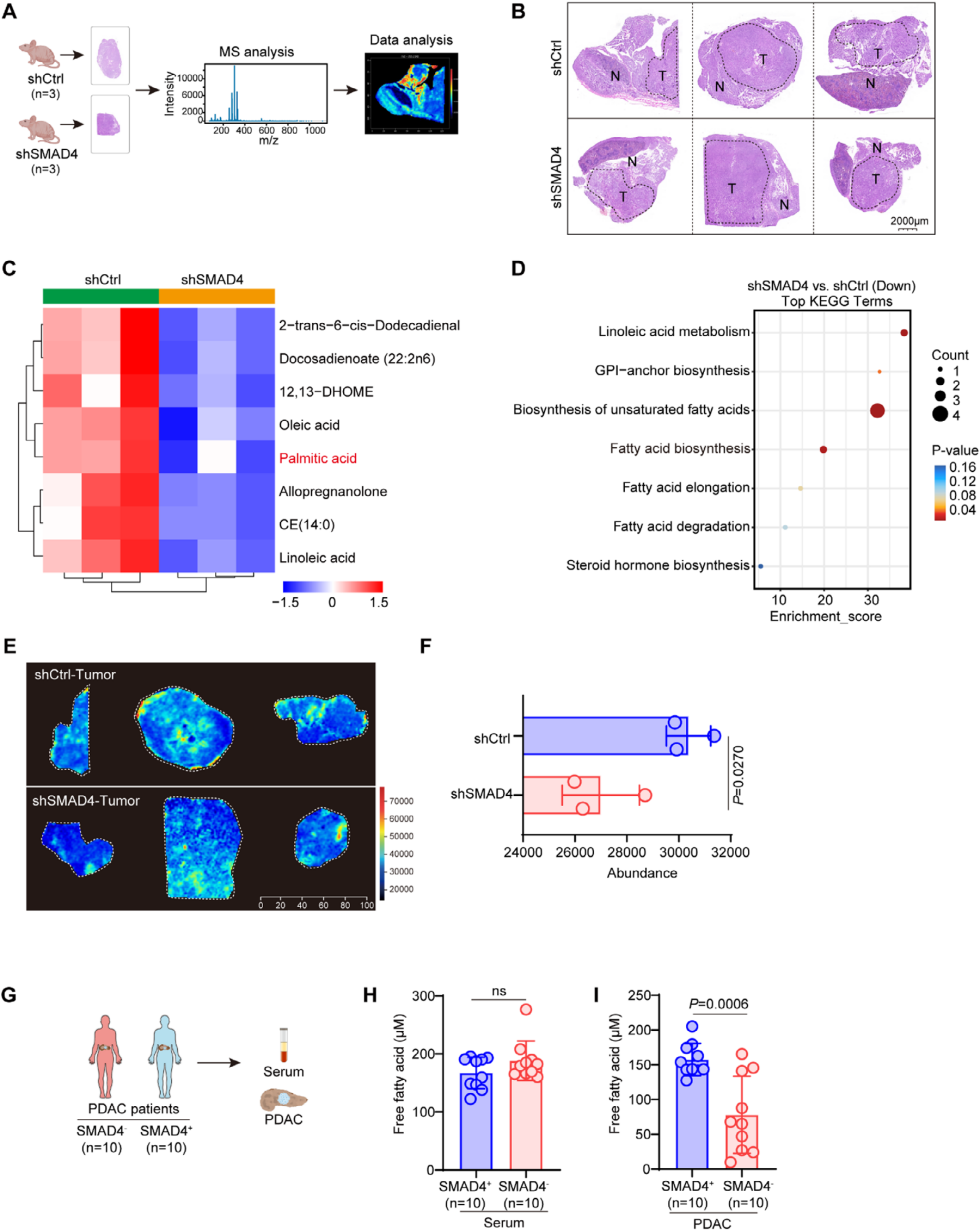
Spatial metabolomics reveals that SMAD4 deficiency decreases palmitic acid. (A) Schematic workflow of spatial metabolomics analysis performed on tumor tissues derived from shCtrl and shSMAD4 groups (n = 3). (B) Representative H&E staining images of the tumor sections. Dashed lines outline the tumor regions (T) and non‐tumor regions (N). Scale bar, 2000 µm. (C) Heatmap visualizing the differential abundance of lipid metabolites between shCtrl and shSMAD4 tumors. Palmitic acid (marked in red) was significantly downregulated in the shSMAD4 group. (D) KEGG pathway enrichment analysis of the downregulated metabolites in shSMAD4 tumors. The top enriched pathways include biosynthesis of unsaturated fatty acids and fatty acid biosynthesis. (E) Representative spatial distribution images of palmitic acid in shCtrl and shSMAD4 tumor tissues. (F) Quantification of the relative abundance of palmitic acid in the tumor regions shown in (E). (G) Schematic diagram of the clinical validation strategy. Serum and tissue samples were collected from PDAC patients stratified by SMAD4 status (n = 10 per group) for free fatty acid detection. (H) Free fatty acid analysis levels in PDAC patients (SMAD4^−^ vs. SMAD4^+^). (I) Free fatty acid analysis levels in PDAC tumor tissues. SMAD4^+^ tumors exhibited significantly higher free fatty acid levels compared to SMAD4^−^ tumors. Data are presented as mean ± SD. Statistical analyses were performed by Student's t‐test.

To explore the metabolic heterogeneity between SMAD4^+^ and SMAD4^−^ pancreatic cancer, we applied spatial metabolomics to compare the metabolite landscape in wild‐type (shCtrl, n = 3) and SMAD4‐deficient (shSMAD4, n = 3) mouse pancreatic cancer models for analysis (Figure 1A,B). Overall, in the shSMAD4 pancreatic cancer, we identified 8 metabolites that were downregulated (Figure 1C), while 16 metabolites were upregulated (Figure S1A) compared to shCtrl controls. Specifically, we noted that fatty acids including palmitic acid, docosadienoate and 12,13‐DHOME were dramatically reduced upon SMAD4 depletion (Figure 1C). Further KEGG analysis also revealed that the lipid metabolism pathway was significantly affected (Figure 1D and Figure S1B). Palmitic acid, the initial and predominant product of fatty acid synthesis, is the most abundant saturated fatty acid accounting for 20%–30% of total fatty acids in the human body [30, 31, 32]. Therefore, we further investigated the impact of SMAD4 expression on fatty acid biosynthesis, with a specific focus on palmitic acid. The spatial distribution and abundance in palmitic acid were significantly reduced, specifically within the tumor regions of shSMAD4 samples (Figure 1E,F).

Furthermore, we collected serum samples from healthy individuals (n = 20) and PDAC patients (n = 20) and subjected them to untargeted metabolomics mass spectrometric analysis (Figure S1C,D). Despite the absence of detectable palmitic acid in these 40 samples, we revealed a metabolic serum landscape in both healthy individuals and PDAC patients. Additionally, lipid metabolism dysregulation was also observed, such as biosynthesis of unsaturated fatty acids (Figure S1E) and involving metabolites like arachidonoylcarnitine (Figure S1F), suggesting the metabolic reprogramming of lipid metabolism for PDAC patients.

At last, we analyzed the free fatty acid levels in serum and PDAC tumor tissues in SMAD4 low expression (SMAD4^−^) (n = 10) and SMAD4 high expression (SMAD4^+^) PDAC patients (n = 10) (Figure 1G). Interestingly, despite no dramatic difference of free fatty acid levels in serum between the SMAD4^−^ and SMAD4^+^ PDAC patients (Figure 1H), the free fatty acid showed a marked reduction in the tissues of SMAD4^−^ compared to SMAD4^+^ tumors (Figure 1I), which was consistent with the findings from spatial metabolomics examination.

Collectively, these findings suggest that tumoral SMAD4 expression contributes to the synthesis of fatty acid levels in PDAC tissues, whereas the palmitic acids were reduced upon SMAD4 inactivation.

### 3.2 SMAD4 Promotes FASN‐Dependent Palmitic Acid Biosynthesis in PDAC

**FIGURE 2:**
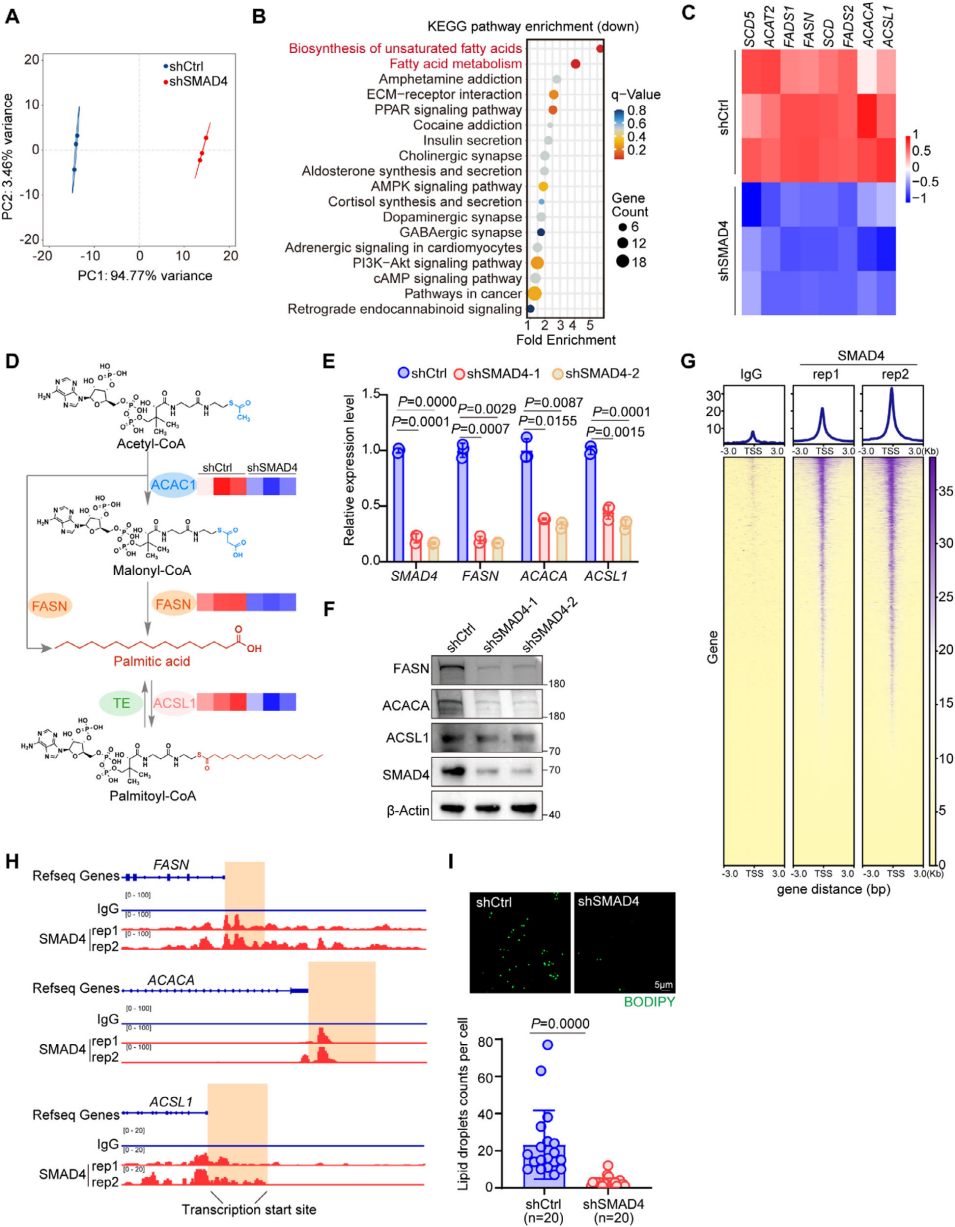
SMAD4 promotes FASN‐dependent palmitic acid biosynthesis in PDAC. (A) Principal component analysis (PCA) plot of RNA‐seq data derived from shCtrl (n = 3) and shSMAD4 (n = 3) cells (|log_2_(FC)|>1, q‐Value< 0.05). (B) KEGG pathway enrichment analysis of the downregulated genes in shSMAD4 cells. Displaying the top enriched pathways with the smallest q‐values. (C) Heatmap depicting the relative expression levels of differential genes involved in lipid metabolism. (D) Schematic diagram of the fatty acid synthesis pathway. The accompanying mini‐heatmaps illustrate the downregulated expression of key enzymes (ACACA, FASN, ACSL1) in shSMAD4 cells compared to shCtrl. (E) qRT‐PCR analyses validating the mRNA expression levels of *SMAD4*, *FASN*, *ACACA*, and *ACSL1* in shCtrl and shSMAD4 cells. (F) Western blot analyses confirming the protein expression levels of SMAD4, FASN, ACACA, and ACSL1 in shCtrl and shSMAD4 cells. (G) Average profiles of SMAD4 binding and IgG control peak densities centered at transcription start sites (TSS), as determined by CUT&Tag assays. (H) Representative Integrative Genomics Viewer (IGV) tracks showing SMAD4 binding peaks at the promoter regions of *FASN*, *ACACA*, and *ACSL1*. (I) Representative images of BODIPY staining (up) and quantification of lipid droplets per cell (down) in shCtrl and shSMAD4 cells. Scale bar, 5 µm. Data are presented as mean ± SD. Statistical analyses were performed by Student's t‐test or two‐way ANOVA.

To explore the potential mechanism by which SMAD4 functions in PDAC, we performed RNA sequencing analysis (RNA‐seq) to identify transcriptional alterations following SMAD4 deletion [4](Figure 2A). Compared to shCtrl cells, shSMAD4 cells revealed 619 downregulated and 901 upregulated genes. KEGG pathway analysis further indicated that SMAD4 depletion markedly reduced the activity of lipid metabolism (Figure 2B and Figure S2A). In the shSMAD4 group, the transcription of the key genes related to fatty acid metabolism and biosynthesis was significantly reduced such as *SCD5* and *FASN* (Figure 2C). Among them, FASN and ACSL1 are two enzymes responsible for palmitic acid and palmitoyl‐CoA synthesis, respectively (Figure 2D). To validate this, we employed both qRT‐qPCR and western blotting to assess the impact of SMAD4 knockdown on the *FASN*, *ACACA*, and *ACSL1*. Consistent with our RNA‐seq data, SMAD4 depletion led to a significant and simultaneous reduction in the mRNA (Figure 2E) and protein (Figure 2F) levels of all three genes.

To further investigate the transcriptional regulatory role of SMAD4 in PDAC cells, we performed CUT&Tag assays at the whole‐genome level. TSS (transcription start site) enrichment was strengthened following the SMAD4 enrichment (Figure 2G), and the observed peak differences were predominantly localized to the promoter regions (Figure S2B). GO and KEGG analyses further revealed the SMAD4‐enriched pathways (Figure S2C,D). These findings were consistent with previous studies demonstrating that SMAD4 acts as a key transcription factor in the TGF‐β signaling pathway, regulating the expression of TGF‐β‐responsive genes such as SMAD7, SnoN and plasminogen activator inhibitor 1 (PAI‐1) [33]. The CUT&Tag assay results confirmed the direct binding of SMAD4 to the promoters of *FASN*, *ACACA* and *ACSL1* (Figure 2H). After that, we performed a more targeted CUT&RUN assay followed by qPCR. This experiment confirmed a significant enrichment of SMAD4 binding on the promoters of *FASN* and *ACSL1* in PDAC cells, suggesting that SMAD4 is a direct transcriptional regulator of *FASN* and *ACSL1* (Figure S2E). However, the lack of SMAD4 enrichment at *ACACA* promoter indicates an indirect regulatory manner. Given that these genes are crucial enzymes involved in the synthesis of long‐chain saturated fatty acids, specifically palmitic acid, we further observed decreased lipid in shSMAD4 cells compared with that in shCtrl cells, as evidenced by BODIPY staining (Figure 2I).

Taken together, our results demonstrate that SMAD4 plays a crucial role in upregulating the expression of key lipogenic genes in PDAC cells, acting as a direct transcriptional regulator for *FASN* and *ACSL1*, which thereby influencing fatty acid production and shedding light on metabolic changes in pancreatic cancer.

### 3.3 SMAD4 is Palmitoylated at Cys363

**FIGURE 3:**
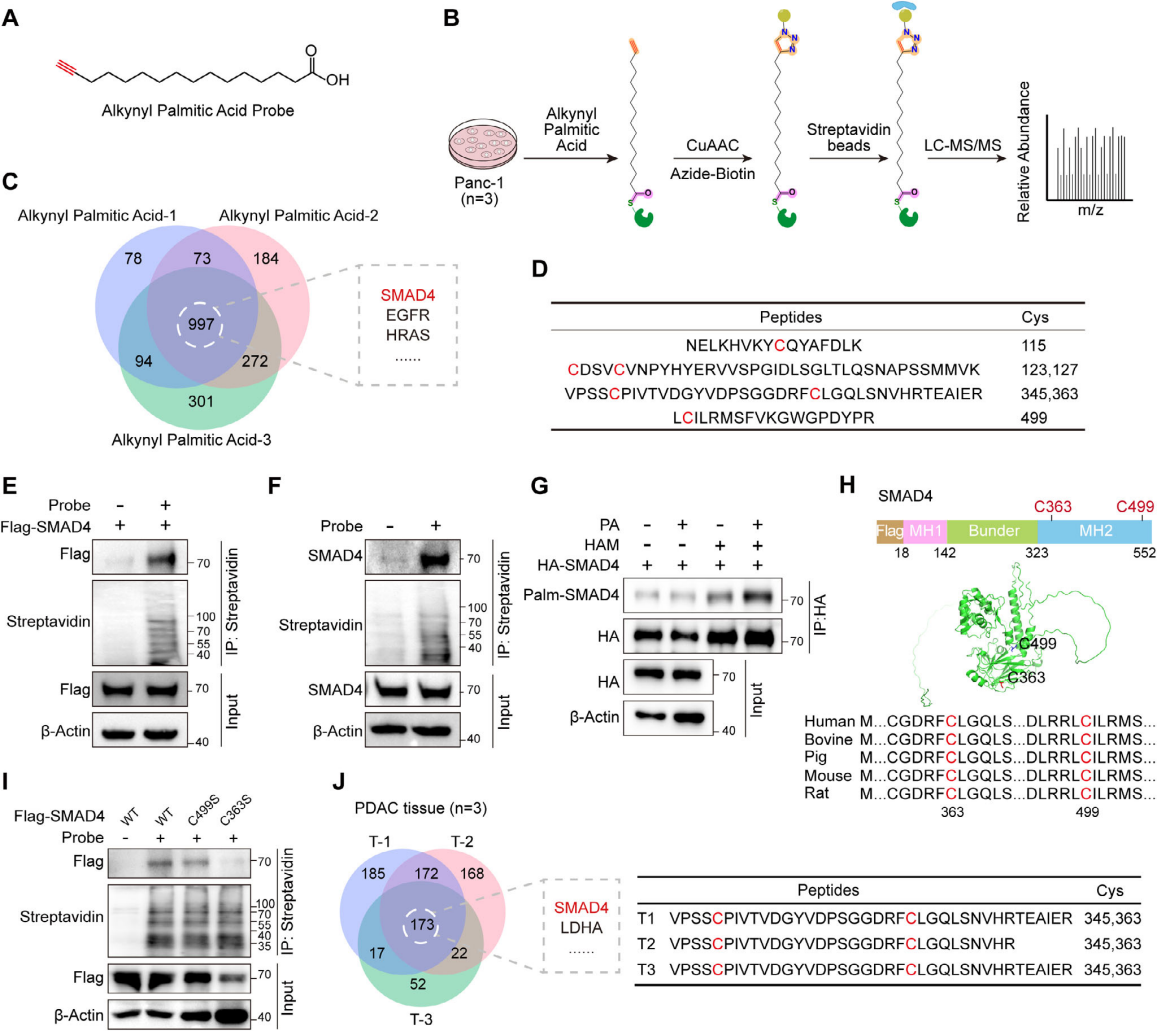
SMAD4 is palmitoylated at Cys363. (A) Chemical structure of alkynyl palmitic acid probe used for metabolic labeling. (B) Schematic workflow for labeling substrate proteins using the click chemistry method. Panc‐1 cells were labeled with the alkynyl‐palmitic acid probe, reacted with biotin‐azide via CuAAC, enriched by streptavidin beads, and analyzed by LC‐MS/MS. (C) Venn diagram showing the overlap of identified palmitoylated proteins from three biological replicates. SMAD4 was consistently identified in all three replicates. (D) List of peptides of SMAD4 identified by mass spectrometry in Panc‐1 cells. The detected cysteine residues are highlighted. (E) Western blot analyses validating exogenous Flag‐SMAD4 palmitoylation by the click chemistry‐based assay in HEK293FT cells. (F) Western blot analyses validating endogenous SMAD4 palmitoylation using the click chemistry‐based assay in Panc‐1 cells. (G) Validation of SMAD4 palmitoylation using the Acyl‐Biotin Exchange (ABE) assay in HEK293FT cells. (H) Structural characterization of SMAD4: domain architecture of SMAD4 highlighting potential cysteine sites (up), 3D structure showing the spatial location of Cys363 and Cys499 (middle), sequence alignment across different species demonstrating the evolutionary conservation of Cys363 and Cys499 (bottom). (I) Western blotting analysis to identify the SMAD4‐specific palmitoylation site by the click chemistry‐based assay, with HEK293FT cells transfected with plasmids encoding wild‐type SMAD4 or its cysteine mutants. (J) Venn diagram showing the overlap of palmitoylated proteins identified in PDAC tumor tissues (n = 3). The table on the right confirms the detection of the Cys363‐containing peptide in patient samples.

Palmitic acid is the most abundant saturated fatty acid in the daily diet and has been reported to serve as the substrate for protein palmitoylation [34]. Palmitic acid is converted to palmitoyl‐CoA and then links cysteine residues to proteins through thioester bonds in a reversible process, which is also called S‐palmitoylation. Therefore, we hypothesized that SMAD4 undergoes palmitoyl‐modification in SMAD4‐wildtype pancreatic cancer. To investigate this, we utilized the alkynyl palmitic acid as a metabolic probe (Figure 3A), and performed the proteomic analysis of palmitoylated proteins in Panc‐1 cells (Figure 3B). Notably, this analysis revealed that SMAD4 is a palmitoylated protein (Figure 3C,D). To validate this finding, we employed two palmitoyl‐conjugation assays, including the click chemistry reaction to biotin‐azide assay and acyl‐biotin exchange (ABE) palmitoylation assay. Palmitoylation of either exogenously expressed Flag‐SMAD4 in HEK293FT cells (Figure 3E) or endogenous SMAD4 in Panc‐1 (Figure 3F) was confirmed by using streptavidin to detect SMAD4 protein labeled through click chemistry. Additionally, the ABE assay further confirmed SMAD4 palmitoylation in HEK293FT cells by using streptavidin to detect SMAD4 protein labeled [35, 36] (Figure 3G). Taken together, these results demonstrated that both ectopically expressed and endogenous SMAD4 undergo palmitoylation.

Next, we aimed to identify the cysteine residues of S‐palmitoylated SMAD4. Combining the proteomic analysis of peptides with the motif‐based prediction tool GPS‐Palm, we predicted two potential single palmitoylation sites in SMAD4 (Cys363, Cys499) (Figure 3H) [17, 36]. To pinpoint the functional palmitoylation sites on SMAD4, we individually mutated each of the two conserved cysteine residues in Flag‐tagged SMAD4 to serine or alanine and assessed the palmitoylation status of these mutants [16, 37]. Using the click chemistry method, we found that the palmitoylation of SMAD4 was almost completely abolished when C363 was mutated (Figure 3I).

Furthermore, we also applied chemoproteomic profiling to examine the presence of palmitoylated SMAD4 in PDAC tissues. Briefly, fresh human PDAC tissue samples were metabolically labeled with alkynyl‐palmitic acid and subjected to chemoproteomic analysis (Figure S3A). This approach successfully identified known palmitoylated proteins such as LDHA [38], confirming the validity of our method (Figure 3J). Importantly, palmitoylated SMAD4 was reproducibly identified across tumor tissues with peptides harboring Cys363 residue (Figure 3J and Figure S3B).

Thus, these findings strongly indicate that C363 is a critical palmitoylation site on SMAD4.

### 3.4 SMAD4 Palmitoylation Enhances Its Protein Stability and Facilitates Interaction with Nuclear Importins

**FIGURE 4:**
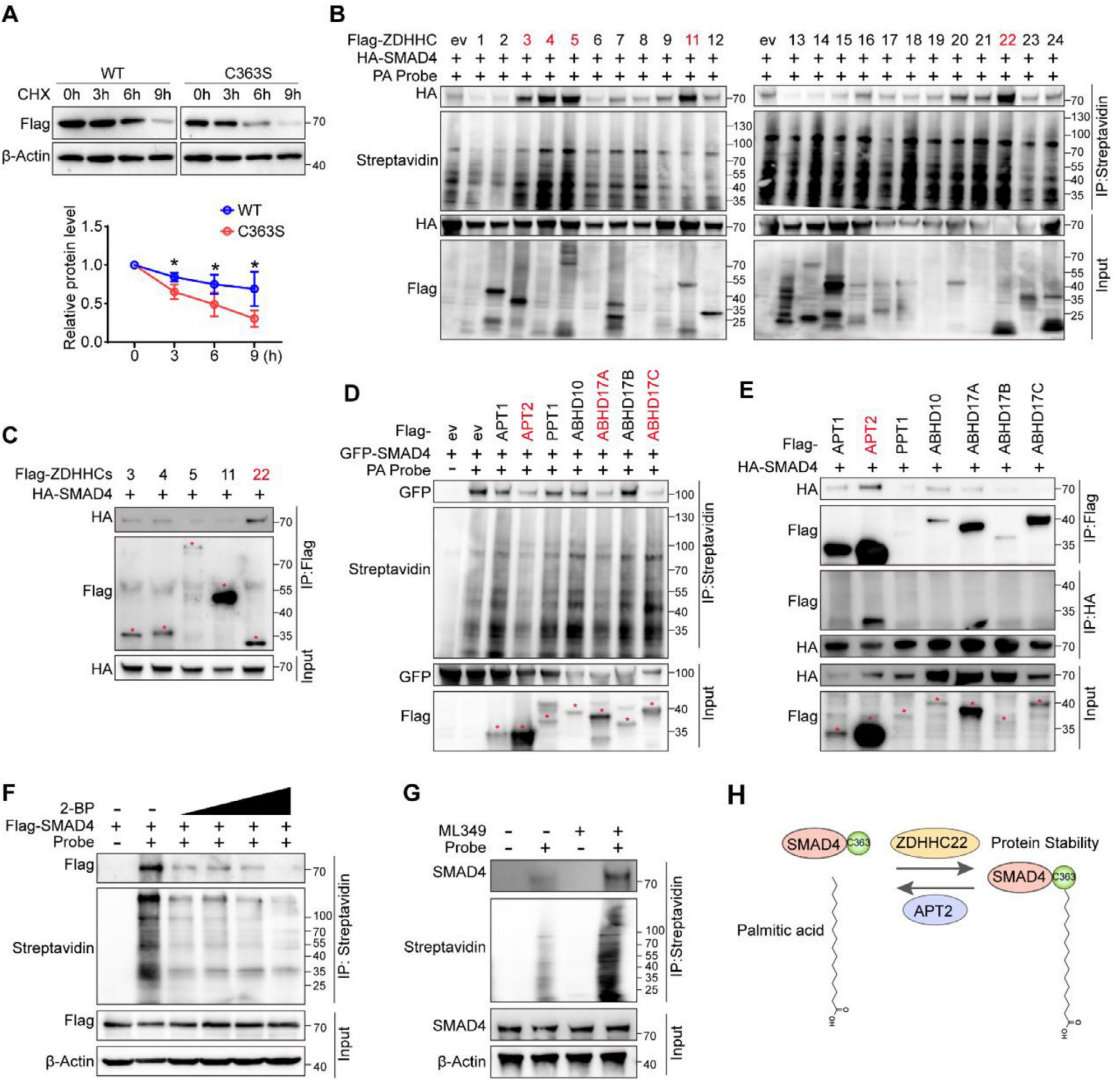
ZDHHC22 and APT2 reversibly regulate SMAD4 palmitoylation. (A) Cycloheximide (CHX) chase assay analyzing the protein stability of wild‐type (WT) and mutant (C363S) SMAD4. The quantification of SMAD4 protein levels is shown below. Data are presented as mean ± SD. Statistical analyses were performed by two‐way ANOVA. (B) Screening of ZDHHC PATs that promote SMAD4 palmitoylation. HEK293FT cells were co‐transfected with HA‐SMAD4 and Flag‐tagged ZDHHCs, followed by click chemistry‐based detection. ZDHHC3, 4, 5, 11, and 22 (marked in red) significantly enhanced SMAD4 palmitoylation. (C) Co‐IP assay validating the interaction between SMAD4 and the candidate ZDHHCs. ZDHHC22 showed the strongest interaction with SMAD4. (D) Screening of depalmitoylases involved in SMAD4 depalmitoylation. Cells were co‐transfected with Flag‐tagged depalmitoylases and GFP‐SMAD4. Overexpression of APT2 (marked in red) markedly reduced SMAD4 palmitoylation levels. (E) Co‐IP and Western blotting analysis validating the physical interaction between HA‐SMAD4 and APT2 in HEK293FT cells. (F) Western blot analysis demonstrating that treatment with the palmitoylation inhibitor 2‐Bromopalmitate (2‐BP) induces a dose‐dependent decrease in SMAD4 palmitoylation. (G) Western blotting analysis demonstrating that treatment with the APT2 inhibitor (ML349) increases SMAD4 palmitoylation levels. (H) Proposed model for the reversible palmitoylation of SMAD4. ZDHHC22 acts as the "writer" to attach palmitate to Cys363, while APT2 acts as the "eraser" thereby dynamically regulating SMAD4 protein stability.

We next performed a set of evaluation assays to address the function of the SMAD4 palmitoylation. First, we performed a cycloheximide (CHX) assay to monitor the protein turnover rates of the wild and SMAD4^C363S^ mutant proteins. In contrast to the rapid degradations of the SMAD4^C363S^ mutant protein, the wildtype SMAD4 group showed much slower turnover rates (Figure 4A). In addition, SMAD4^C363S^ mutant ubiquitination increases, which indicates the cause of the change in stability (Figure S3C). Moreover, supplementation with PA significantly increased the half‐life of wildtype SMAD4, while PA treatment failed to accelerate half‐life of the C363S mutant, which reflects the loss of SMAD4 stability in the C363S mutant is specifically caused by the absence of palmitoylation (Figure S3D). Then, we used AlphaFold for prediction and Pymol for structural comparison. The folding between the wild and SMAD4^C363S^ mutant remained largely unchanged (Figure S3E). Next, we performed a co‐IP assay to study the binding of the SMAD2/3/4 complex (Figure S3F). Formation of the SMAD2/3/4 complex was barely affected by the mutation of 363. Additionally, as importin 7 and 8 have been reported to be required for nuclear import of SMAD4 [39], we thus performed co‐IP experiments to examine their interactions with SMAD4^WT^ or SMAD4^C363S^ in PDAC cells. Results clearly demonstrated that the SMAD4^WT^ could efficiently bind to both importin 7 and 8, while such interaction with importin 7 was greatly attenuated in SMAD4^C363S^ mutant, indicating that SMAD4 palmitoylation is a requirement step for subsequent importins‐mediated nuclear translocation. (Figure S3G).

Overall, these new evidences indicated that SMAD4 palmitoylation is essential for its protein stability maintenance and nuclear import, but does not affect its conformational changes and assembly for the SMAD2/3/4 complex formation (Figure S3H).

### 3.5 ZDHHC22 and APT2 Reversibly Regulate SMAD4 Palmitoylation

To identify the specific protein S‐acyltransferases responsible for SMAD4 palmitoylation, we overexpressed 23 Flag‐tagged ZDHHC palmitoyl acyltransferases along with HA‐tagged SMAD4 in HEK293FT cells and then performed a click chemistry‐based assay to detect SMAD4 palmitoylation. Among these, overexpression of ZDHHC3, 4, 5, 11 and 22 significantly enhanced SMAD4 palmitoylation, with ZDHHC22 showing the most prominent effect (Figure 4B). Furthermore, co‐IP experiments confirmed the interaction between ZDHHC22 and SMAD4, supporting their functional association (Figure 4C).

To determine the enzyme responsible for the depalmitoylation of SMAD4, we overexpressed seven candidate Flag‐tagged depalmitoylases along with HA‐tagged SMAD4 in HEK293FT cells and assessed SMAD4 palmitoylation levels using a click chemistry‐based assay. Among these, our results demonstrated a decrease in SMAD4 palmitoylation following APT2, ABHD17A and ABHD17C overexpression (Figure 4D). Moreover, this interaction between APT2 and SMAD4 was further confirmed through co‐IP experiments (Figure 4E).

In addition, supporting our hypothesis, treatment with 2‐bromopalmitate (2‐BP), a widely used inhibitor of general protein palmitoylation [16, 17], substantially reduced SMAD4 palmitoylation (Figure 4F), whereas ML349 increased, SMAD4 palmitoylation (Figure 4G).

Collectively, these results indicated that among the ZDHHC family members tested, ZDHHC22 plays a crucial role in mediating the palmitoylation of SMAD4, and established APT2 as the primary regulatory enzyme responsible for SMAD4 depalmitoylation (Figure 4H).

### 3.6 Palmitic Acid–SMAD4 Palmitoylation–FASN Forms a Positive Feedback Loop Drives PDAC

**FIGURE 5:**
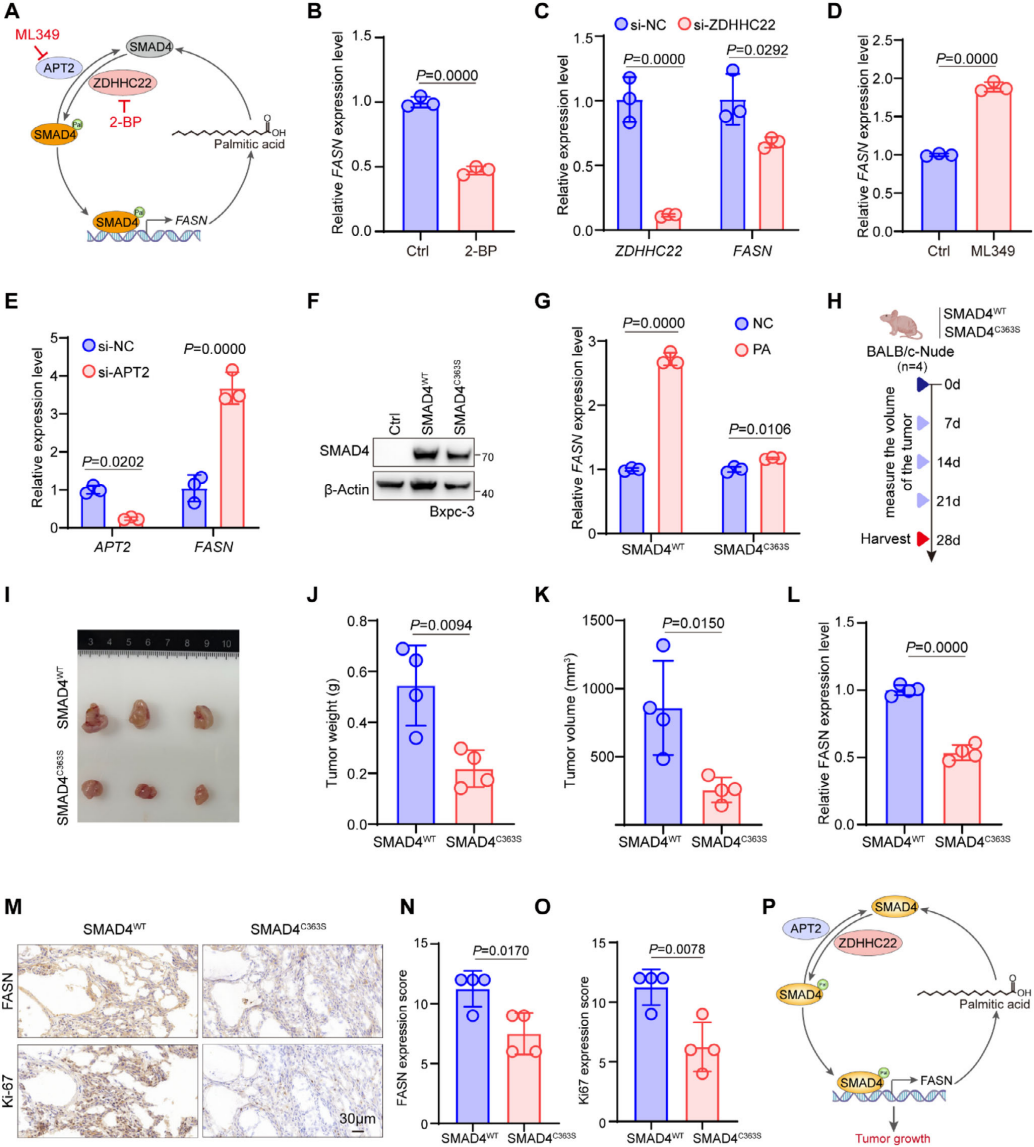
PA–SMAD4 palmitoylation–FASN forms a positive feedback loop drives PDAC. (A) Schematic diagram illustrating the ZDHHC22‐SMAD4‐APT2 regulatory loop involving 2‐BP (palmitoylation inhibitor) and ML349 (APT2 inhibitor). (B‐E) qRT‐PCR analysis of *FASN* mRNA expression in cells treated with 2‐BP (B), *ZDHHC22* siRNA (C), ML349 (D), or *APT2* siRNA (E). (F) Western blot validating the construction of stable Bxpc‐3 cell lines expressed Myc‐SMAD4 WT and C363S mutant. (G) qRT‐PCR analysis of *FASN* expression in SMAD4 WT and C363S mutant‐expressing cells treated with or without palmitic acid (PA). PA stimulation failed to upregulate FASN in the C363S mutant group. (H) Experimental design for the in vivo mouse model. Cells stably expressing SMAD4 WT or C363S mutant were injected subcutaneously into nude mice (n = 4 per group). (I) Representative images of dissected tumors from the SMAD4‐WT and C363S mutant groups. (J,K) Quantification of tumor weight (J) and tumor volume (K) of SMAD4 WT or C363S mutant groups. The C363S mutation significantly suppressed tumor growth. (L) qRT‐PCR analysis of *FASN* expression in the excised tumor tissues. (M) Representative IHC staining of FASN and Ki‐67 in tumor tissue from each group. Scale bar, 30 µm. (N,O) Quantification of the IHC staining scores for FASN (N) and Ki67 (O). (P) Schematic diagram illustrating the palmitic acid‐SMAD4 palmitoylation‐FASN regulatory loop in pancreatic cancer. ZDHHC22‐mediated palmitoylation stabilizes SMAD4, promoting its transcriptional regulation of FASN and driving tumor growth, a process dynamically reversed by APT2. Data are presented as mean ± SD. Statistical analyses were performed by Student's *t*‐test or two‐way ANOVA.

To elucidate the role of SMAD4 palmitoylation in driving target gene transcription and PDAC development, we applied both pharmacological inhibition and genetic knockdown approaches of the ZDHHC22 and APT2 to modulate SMAD4 palmitoylation and assess the corresponding effect on *FASN* transcription (Figure 5A). On one hand, treatment with 2‐BP, a stable analog of palmitate known for inhibiting various lipid metabolism‐related enzymes and protein palmitoylation [16, 17], markedly reduced *FASN* transcription (Figure 5B). Similarly, knockdown of ZDHHC22 phenocopied to reduce *FASN* mRNA expression (Figure 5C). In sharp contrast, pharmacological inhibition of APT2 with ML349 (Figure 5D) or genetic silencing (Figure 5E) dramatically stimulated *FASN* transcription. Furthermore, we directly overexpressed wild‐type SMAD4 (SMAD4^WT^) and the non‐palmitoylatable SMAD4‐C363S mutant (SMAD4^C363S^) in Bxpc‐3 cells (Figure 5F). When palmitic acid was added, compared with SMAD4^WT^, SMAD4^C363S^ failed to upregulate FASN expression (Figure 5G). In conclusion, our findings support the existence of a positive feedback loop formed by SMAD4, FASN, and palmitic acid.

Next, we further examined the function of SMAD4 palmitoylation in subcutaneous tumor models (Figure 5H). Compared with SMAD4^WT^ tumors, the non‐palmitoylatable SMAD4^C363S^ group showed much smaller tumor size, indicating that SMAD4 palmitoylation functions to promote tumor growth (Figure 5I–K). In line with the positive feedback loop notion, the mRNA level of *FASN* in SMAD4^C363S^ group was significantly lower than SMAD4^WT^ tumors (Figure 5L). Moreover, immunohistochemical analysis of FASN and Ki‐67 was also consistent with above results detected (Figure 5M), and found that both FASN (Figure 5N) and Ki‐67 levels (Figure 5O) were higher in SMAD4^WT^ groups than in SMAD4^C363S^ groups.

Collectively, these results indicate that palmitic acid–SMAD4 palmitoylation–FASN forms a positive feedback loop to drive tumorigenesis in pancreatic cancer (Figure 5P).

### 3.7 SMAD4 Palmitoylation Renders Pancreatic Cancer Cells Sensitive to Radiotherapy

**FIGURE 6:**
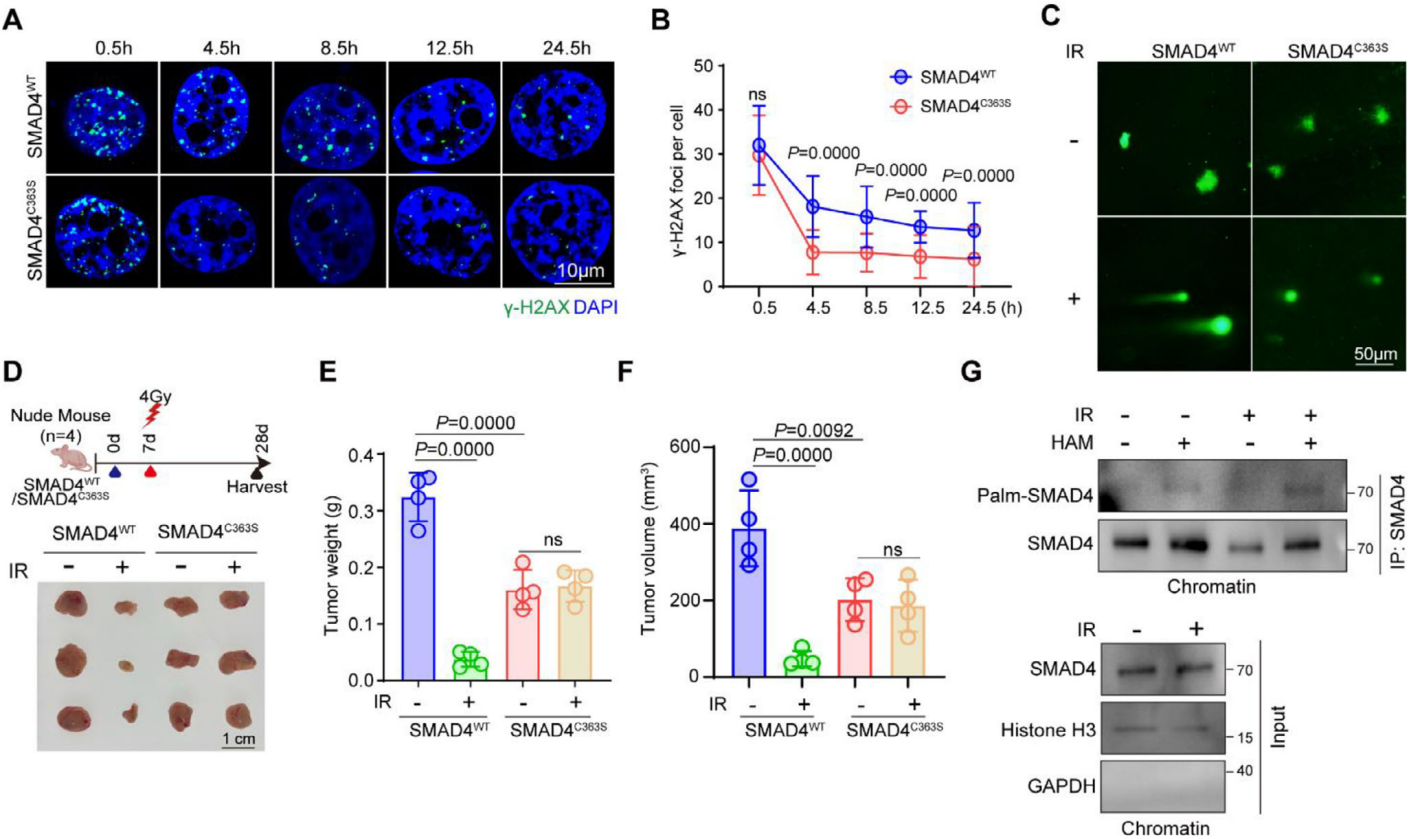
SMAD4 palmitoylation renders pancreatic cancer cells sensitive to radiotherapy. (A) Representative immunofluorescence images of γ‐H2AX foci (green) in SMAD4^WT^ and SMAD4^C363S^ mutant expressing cells at the indicated time points after irradiation (IR, 10 Gy). Scale bar, 10 µm. (B) Quantification of γ‐H2AX foci per cell from the experiment in (A). The C363S mutant cells showed significantly faster clearance of DNA damage foci compared to WT cells. (C) Representative images of the neutral comet assay evaluating DNA damage in cells treated with or without IR. (D) Schematic diagram of the experimental design for the in vivo radiosensitivity study in nude mice (n = 4). Representative images of subcutaneous tumors derived from cells stably expressing SMAD4^WT^ or SMAD4^C363S^ mutant. Mice were treated with or without IR (n = 4 per group). (E) Quantification of tumor weight of SMAD4^WT^ or SMAD4^C363S^ mutant groups treated with or without IR. IR treatment significantly reduced tumor burden in the SMAD4‐WT group but failed to inhibit tumor growth in the SMAD4 C363S mutant group. (F) Quantification of tumor volumes of SMAD4^WT^ or SMAD4^C363S^ mutant groups treated with or without IR. (G) Detection of palmitoylated SMAD4 in the chromatin fraction. Chromatin‐bound proteins were isolated, and S‐palmitoylation was assessed using the ABE assay. IR treatment induced the recruitment of palmitoylated SMAD4 to the chromatin. Data are presented as mean ± SD. Statistical analyses were performed by Student's t‐test or two‐way ANOVA.

SMAD4, which previous studies have shown shuttles between the cytoplasm and nucleus, accumulates in the nucleus following TGF‐β stimulation or irradiation [4, 33]. Upon activation, SMAD4 binds to SMAD2 and SMAD3 to form a heterodimeric complex and together they translocate to the nucleus to exert transcriptional functions. Our previous work has demonstrated that the radiomimetic chemical NCS enhances SMAD4 nuclear translocation and fosters its interaction with PARP1, thus SMAD4 attenuates PARP1‐mediated DNA repair and renders pancreatic cancer cells sensitive to radiotherapy [4]. To explore how SMAD4 palmitoylation modulates its function on radiotherapy, we monitored the IR‐induced foci formation of DNA damage markers such as γ‐H2AX, each at various time points, and the results of this monitoring revealed that the damaged DNA was repaired much more rapidly in SMAD4^C363S^ cells than in SMAD4^WT^ cells (Figure 6A,B), suggesting an enhanced DNA repair capability. Additionally, we performed a neutral comet assay to validate the roles of candidate hits in DNA repair (Figure 6C and Figure S4A). Consistent with the γ‐H2AX data, the SMAD4^C363S^ mutation substantially increased the rate of DNA repair compared to the WT control group. Together, these direct functional assays demonstrate that preventing SMAD4 palmitoylation at Cys363 accelerates DNA damage repair.

Next, we further examined the therapeutic responses to radiotherapy on therapeutic responses in subcutaneous tumor models (Figure 6D‐F). The SMAD4^WT^ group exhibited a notable decrease in tumor size after radiotherapy, which was consistent with our previous research [4, 6]. We also observed that the tumor weights and volumes of the SMAD4^C363S^ group were lower than those of the SMAD4^WT^ group. In contrast, in the SMAD4^C363S^ groups, mouse models showed no significant differences in size or weight between radiotherapy and control groups (Figure 6E,F). Collectively, these results indicate that SMAD4 palmitoylation enhances radiotherapy sensitivity. In SMAD4^C363S^ groups, in which SMAD4 is non‐palmitoylatable, radiotherapy resistance is exacerbated. Moreover, we then found, through the ABE assay, that chromatin‐bound palmitoylated SMAD4 levels increased following irradiation exposure (Figure 6G).

Moreover, to explore the function of ZDHHC22 and APT2‐mediated SMAD4 palmitoylation, we first performed cell viability analysis that ML349 increases chemotherapy sensitivity (Figure S4B). These results are consistent with the function of SMAD4 palmitoylation.

In summary, these results demonstrate that SMAD4 palmitoylation renders pancreatic cancer cells sensitive to radiotherapy.

### 3.8 The R361H/C Mutants Potentiate the SMAD4 Palmitoylation–FASN Positive Feedback Loop

**FIGURE 7:**
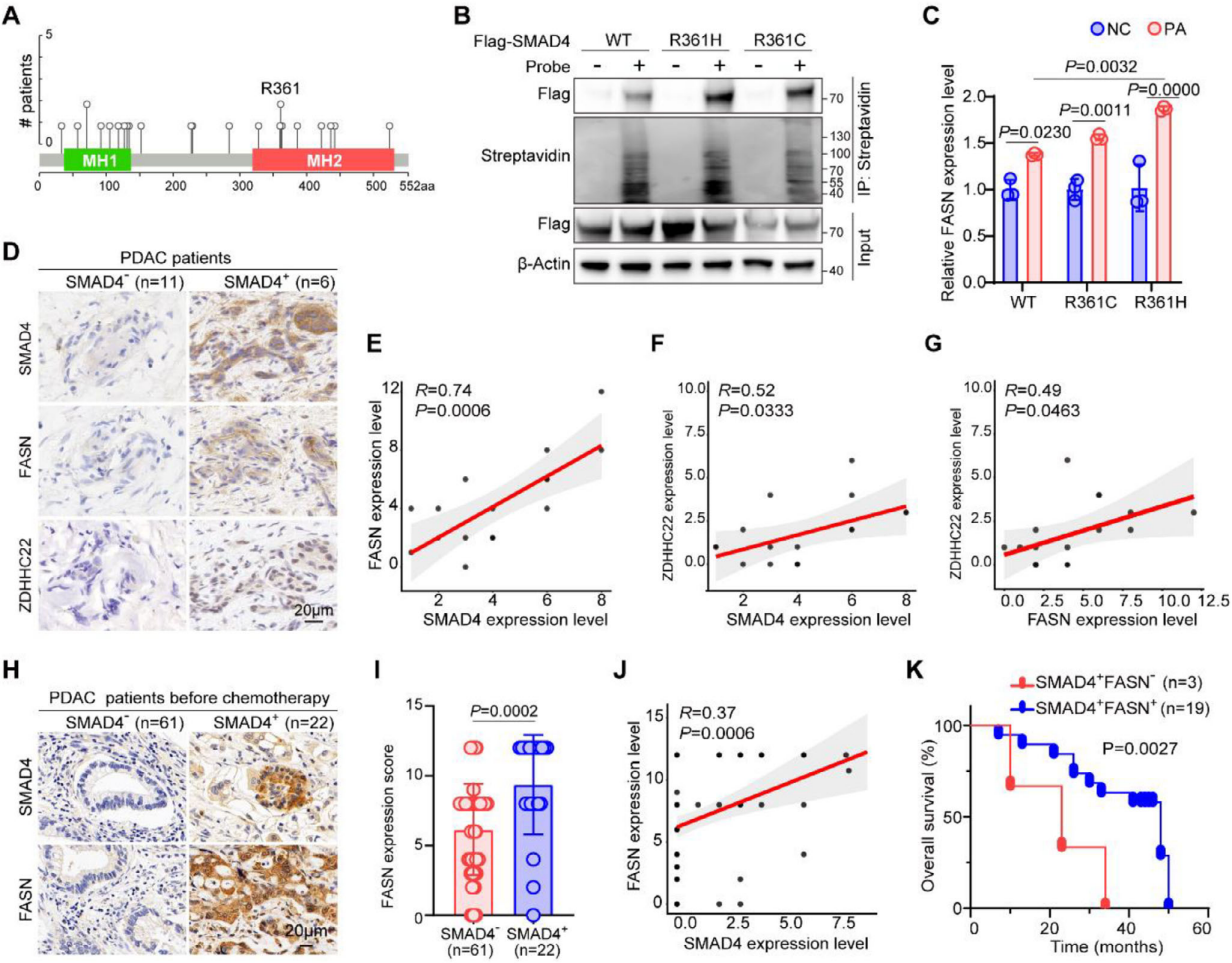
Clinical relevance of SMAD4–FASN axis in PDAC. (A) Lollipop plot displaying the distribution and frequency of *SMAD4* somatic mutations in pancreatic cancer patients from the TCGA database and cBioportal database. R361 is identified as a hotspot mutation site within the MH2 domain. (B) Western blotting analysis of palmitoylation levels in WT SMAD4 and mutant (R361H, R361C) Flag‐SMAD4. R361 mutants exhibited enhanced palmitoylation compared to WT. (C) qRT‐PCR analysis of *FASN* mRNA expression in cells expressing WT or mutant SMAD4, treated with or without PA. The R361 mutants induced significantly higher FASN expression levels. (D) Groups of PDAC patient stratification based on SMAD4 expression status. Representative IHC staining images of SMAD4, FASN, and ZDHHC22 in PDAC tumor tissues. Scale bar, 20 µm. (E‐G) Scatter plots showing the positive correlations between the expression levels of SMAD4 and FASN (E), SMAD4 and ZDHHC22 (F), and FASN and ZDHHC22 (G) in patient tissues. (H) Groups of the clinical cohort used for survival analysis, involving PDAC patients treated with chemotherapy. Representative IHC staining of FASN in SMAD4^−^ and SMAD4^+^ tumor tissues. (I) Quantification of FASN expression scores in the SMAD4^+^ (n = 22) and SMAD4^−^ (n = 61) groups. SMAD4^+^ tumors displayed significantly higher FASN levels. (J) Scatter plots showing the positive correlations between the expression levels of SMAD4 and FASN in patient tissues. (K) Kaplan‐Meier curves for overall survival (OS) of PDAC patients treated with chemotherapy stratified by SMAD4 and FASN expression status. Data are presented as mean ± SD. Statistical analyses were performed by Student's *t*‐test or two‐way ANOVA.

As a key driver mutation, SMAD4 functional loss characterizes multiple gastrointestinal neoplasms, such as appendiceal and colorectal cancer, and with the highest frequency of occurrence observed in PDAC [4, 24, 40, 41]. As shown in the cBioPortal database, R361, which is immediately adjacent to C363, represents the most frequent mutational site of SMAD4 in PDAC patients (Figure 7A). Notably, the R361 mutation is located in the MH2 domain, which is critical for transcriptional activation and complex formation among SMAD proteins [41, 42]. The SMAD4^R361^ mutation has been reported in colorectal cancer for BVZ and 5‐fluorouracil resistance, its methylation for metastasis and boosting Wnt/β‐Catenin signaling [24, 43, 44].

To directly evaluate the functional impact of the clinically relevant R361H/C mutants, we have now assessed their palmitoylation levels and transcriptional activity relative to SMAD4^WT^. Interestingly, the R361H/C mutants exhibited elevated palmitoylation levels compared to SMAD4^WT^, suggesting a potentially enhanced functional effect (Figure 7B). Consistent with this, the addition of palmitic acid induced a stronger upregulation of *FASN* mRNA expression in cells expressing the R361H/C mutants (Figure 7C). Together, these data indicate that the R361H/C mutations, which increase SMAD4 palmitoylation, augment its transcriptional activity toward FASN. This reinforces the proposed model whereby enhanced SMAD4 palmitoylation amplifies the PA‐SMAD4‐FASN feedback loop.

### 3.9 The SMAD4–FASN Axis Defines a Prognostic Divergence Between Disease Progression and Treatment Response in PDAC

Given that our mechanistic data indicate SMAD4 palmitoylation enhances its protein stability, we hypothesized that the expression levels of the acyltransferase ZDHHC22 and the palmitate producer FASN would positively correlate with SMAD4 protein levels in clinical samples. Accordingly, we performed IHC staining for SMAD4, ZDHHC22, and FASN on human PDAC tissues treated with surgery (Figure 7D). As expected, SMAD4‐high (SMAD4^+^) PDAC tissues exhibited significantly higher protein levels of FASN (Figure S4C) and ZDHHC22 (Figure S4D), compared to SMAD4‐low (SMAD4^−^) tissues (Figure 7D). Further correlation analysis revealed positive and significant correlations between SMAD4 with FASN (R = 0.74, *p* = 0.0006; Figure 7E) and ZDHHC22 (R = 0.52, *p* = 0.0333; Figure 7F), as well as between FASN and ZDHHC22 (R = 0.49, *p* = 0.0463; Figure 7G). These correlation results support the functional relevance of the SMAD4 palmitoylation pathway in human pancreatic cancer.

Meanwhile, to investigate the clinical implications of SMAD4 status and palmitic acid levels, we conducted immunohistochemical (IHC) analyses of SMAD4 and FASN expression in tissue microarrays from 85 PDAC patients treated with chemotherapy (Figure 7H). Consistent with our previous findings, we observed SMAD4 deficiency in about half of these PDAC patients (Figure S4E). The IHC analysis of 85 PDAC patients further revealed that FASN expression in SMAD4^+^ PDAC is significantly higher than that in SMAD4^−^ PDACs (Figure 7I), demonstrating a similar positive correlation between SMAD4 and FASN (R = 0.37, *p* = 0.0006; Figure 7J). Nevertheless, such SMAD4^+^FASN^+^ expression predicts better prognosis in PDAC patients undergoing chemoradiotherapy (Figure 7K), aligning with the functional role of SMAD4‐mediated inhibition of DNA repair capacity.

Taken together, these results highlight the diagnostic and therapeutic potential of targeting palmitoylation and the palmitic acid–SMAD4–FASN signaling axis in PDAC.

## 4 Discussion

**FIGURE 8:**
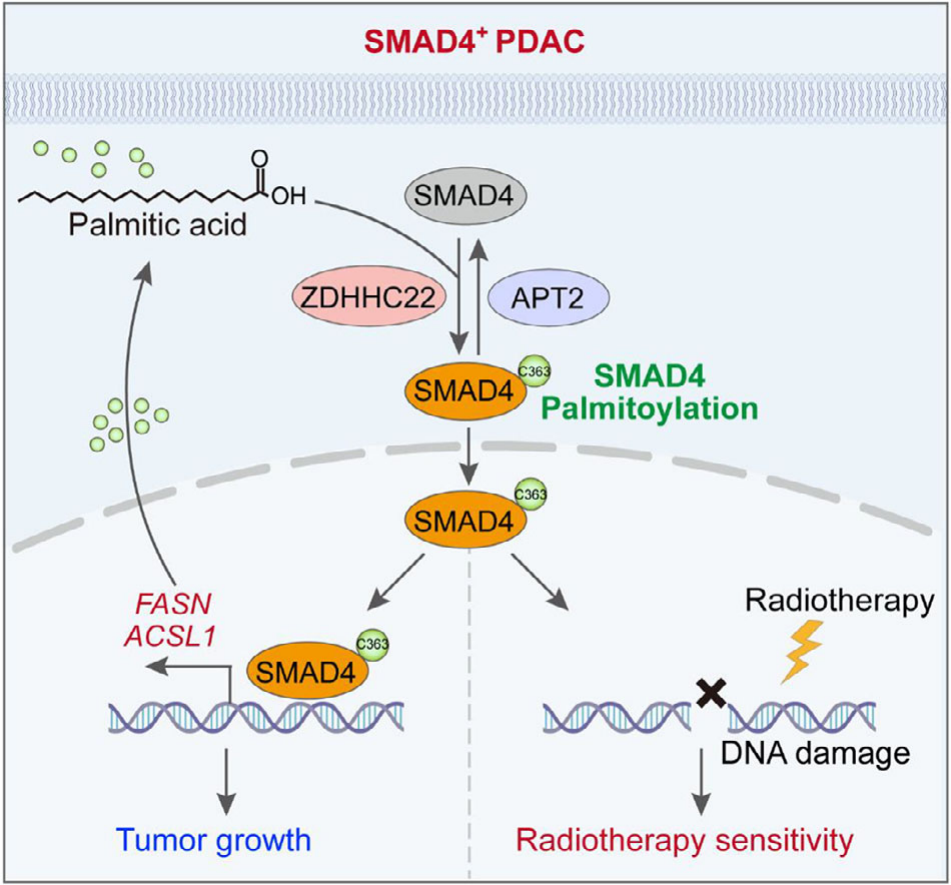
Working model. SMAD4 palmitoylation‐FASN‐palmitic acid feedback loop drives SMAD4 transcriptional activity and eventually promotes pancreatic cancer progression (left panel). Under genotoxic stress (e.g., irradiation), the same palmitoylationinhibits DNA repair, sensitizing cancer cells to radiotherapy (right panel).

Protein posttranslational modifications (PTMs) represent a diverse regulatory layer that shapes chromatin dynamics, gene expression and cellular signaling. Classical PTMs such as acetylation, methylation, and succinylation have been extensively studied in cancer. For instance, lactate‐driven histone lactylation activates ACAT2 transcription [45], while MTHFD1‐mediated decrotonylation promotes pancreatic cancer progression by enhancing ferroptosis resistance [46]. In addition, histone serotonylation remodels lipid metabolism to drive pancreatic cancer tumorigenesis [47]. Collectively, these findings underscore the pivotal role of PTMs in PDAC pathogenesis. In this study, we found that under basal or tumor‐promoting conditions, SMAD4 palmitoylation is driven by elevated local palmitic acid (PA). This modification enhances SMAD4's protein stability, interaction with importins, and transcriptional activity, establishing an elevated PA → SMAD4 Palmitoylation → FASN Transcription positive feedback loop that fuels lipogenesis and tumor growth. Here, palmitoylation confers an oncogenic gain‐of‐function (Figure 8, left panel). Under genotoxic stress (e.g., irradiation), the same palmitoylation‐mediated nuclear accumulation possibly facilitates the interaction of SMAD4 with PARP1, thereby inhibiting PARP1‐dependent DNA repair [4]. In contrast, the deficiency of SMAD4 palmitoylation relieves this suppression in SMAD4^C363S^ cells, consequently making them vulnerable to PARP inhibitors. This palmitoylation impairs the tumor's ability to recover from DNA damage, sensitizing cancer cells to radiotherapy (Figure 8, right panel). In this therapeutic context, the palmitoylated SMAD4 acts to compromise cell survival. Therefore, targeting PA synthesis, SMAD4 palmitoylation, ZDHHC22 or APT2 (2‐BP or specific inhibitors) will provide effective strategies for PDAC therapies (Figure 8).

In recent years, protein palmitoylation has emerged as an equally important PTM in tumor biology. This reversible lipid modification, catalyzed by the ZDHHC family palmitoyl acyltransferases and removed by depalmitoylases, regulates protein stability, subcellular localization, and interaction networks. Palmitoylation has been implicated in diverse oncogenic processes, including tumor progression, the immune environment and drug resistance [17]. In this study, we extend these insights to the TGF‐β signaling axis by demonstrating that SMAD4 undergoes palmitoylation in PDAC, thereby uncovering a previously unrecognized mechanism of SMAD4 regulation.

While palmitoylation of other SMAD2 and SMAD3 has been previously reported, their modification sites (Cys41 and Cys81 in SMAD2; Cys421 in SMAD3) are not conserved in SMAD4 (29, 34) (Figure S4F). We identified Cys363 as a novel palmitoylation site on SMAD4 and functionally characterized its importance. Specifically, SMAD2 S‐palmitoylation promotes phosphorylation of its linker region and TH17 cell differentiation in a mouse model of multiple sclerosis [48]. SMAD3 palmitoylation enhances activation of the TGF‐β signaling pathway, and its interaction with EP300 drives expression of mesenchymal markers in the mesenchymal subtype of GBM [37]. Unlike SMAD2/3 palmitoylation, which modulates phosphorylation and mesenchymal gene expression, SMAD4 palmitoylation at Cys363 specifically enhances its transcriptional activity, linking lipid metabolism to transcriptional regulation in PDAC. This distinction underscores the uniqueness of our findings within the broader SMAD family.

SMAD4 is widely recognized as a tumor suppressor gene, with inactivation occurring approximately 60% of PDAC cases through homozygous deletion or mutation [27]. In pancreatic cancers, approximately 30% patients are found to have homozygous deletion and 20% are identified as inactivation. Our data demonstrate that the prevalent clinical mutations, including R361C and R361H variants, retain the ability to undergo palmitoylation. These findings are significant because they suggest that palmitoylation remains intact in a substantial subset of PDAC patients, including those with wild‐type SMAD4 or palmitoylation‐competent mutants. This observation positions palmitoylation as a potentially druggable vulnerability in PDAC. Targeting SMAD4 palmitoylation, or its regulatory enzymes such as ZDHHC22 and APT2, could therefore represent a viable therapeutic strategy.

Our study also provides functional evidence linking SMAD4 palmitoylation to PDAC biology. We show that SMAD4 palmitoylation regulates the transcription of fatty acid biosynthesis and metabolism enzymes, including FASN and ACSL1, thereby coupling lipid metabolism with transcriptional control. SMAD4 has been reported to regulate the YAP/TAZ‐FASN axis driving lipid metabolism in SMAD4‐deficient PDAC [49]. Also, SMAD4 deficiency also inhibits of lipogenesis in nonalcoholic fatty liver disease [50]. These studies highlight the context‐dependent regulation of lipid metabolism in pancreatic cancer. Our study complements this finding by identifying a distinct, palmitoylation‐dependent mechanism linking SMAD4 to FASN transcriptional regulation in SMAD4‐proficient PDAC tumors. Palmitic acid‐SMAD4 palmitoylation‐FASN generates a positive feedback loop in which palmitic acid promotes SMAD4 palmitoylation, and palmitoylated SMAD4 in turn drives FASN expression and palmitic acid production. Importantly, SMAD4 palmitoylation inhibits DNA repair after irradiation, sensitizing PDAC cells to radiotherapy. These findings suggest that palmitoylation is not only a driver of tumor progression but also a determinant of therapeutic response, providing a rationale for integrating palmitoylation‐targeted strategies with existing treatment modalities.

We acknowledge several limitations. While we mainly employed the click chemistry method and ABE approaches to identify SMAD4 palmitoylation, direct in situ detection of SMAD4 palmitoylation in clinical PDAC tissues remains to be established. Meanwhile, we used FASN and ZDHHC22 expression, and BODIPY staining as indirect readouts of palmitic acid level, which may not fully capture the complexity of lipid metabolism in tumors. More precise mechanisms about SMAD4 palmitoylation affected DNA repair indeed a logical next step, and we plan to conduct further experiments to systematically investigate these mechanisms in our future work. Addressing these limitations mentioned will strengthen the translational relevance of our research findings.

In conclusion, we identify Cys363 as the critical palmitoylation site on SMAD4 and reveal that this modification integrates lipid metabolism with transcriptional control in PDAC. ZDHHC22 and APT2 dynamically regulate SMAD4 palmitoylation, which in turn governs its protein stability, interaction with importins, transcriptional activity and response to radiotherapy. Collectively, our findings establish a SMAD4 palmitoylation‐FASN‐palmitic acid feedback loop as a central regulatory axis in PDAC, and highlight the therapeutic potential of targeting this pathway to improve outcomes in pancreatic cancer treatment.

## Author Contributions

Conceptualization, Y.W., L.A., F.W.; methodology, Y.W., S.Z., J.B., M.J., C.J., X.G., X.L., Y.S., T.Y., Y.H., Y.H., J.Z.; investigation, Y.W., S.Z.; L.A., F.W., T.Y., M.J.; visualization, Y.W., Y.H., S.Z. W.L.; writing – original draft, Y.W., S.Z.; writing – revision, S.Z., J.B., L.A., F.W., Y.Z.; funding acquisition, L.A., F.W.; supervision, J.C., L.A., F.W., Y.Z.

## Funding

This work was supported by the Noncommunicable Chronic Diseases‐National Science and Technology Major Project (2024ZD0533203), Shanghai Oriental Talent program (BJWS2024032), Shanghai Academic/Technology Research Leader (23XD1433000), National Natural Science Foundation of China Grants (82573553), Science and Technology Commission of Shanghai Municipality (23ZR1480400, 23YF1432900), Cultivation Grant for Clinical and Basic Integration Research of Shanghai Tenth People's Hospital (SYYYRH2025021).

## Conflicts of Interest

The authors declare no potential conflicts of interest.

## Supporting information




**Supporting File**: advs74893‐sup‐0001‐SuppMat.docx.

## Data Availability

The data that support the findings of this study are available from the corresponding author upon reasonable request.
