# Supplementary material for: SMAD4 Palmitoylation Drives a Metabolic‐Transcriptional Circuit to Promote Tumorigenesis and Confers Radiosensitivity in Pancreatic Cancer
Source: Adv Sci (Weinh). 2026 Mar 26;13(41):e19791. doi: 10.1002/advs.202519791 (PMC13325641; doi:10.1002/advs.202519791)
Supplement: Supplementary file 1 — Supporting File: advs74893‐sup‐0001‐SuppMat.docx. [file ADVS-13-e19791-s001.docx]

**Supplementary figures**


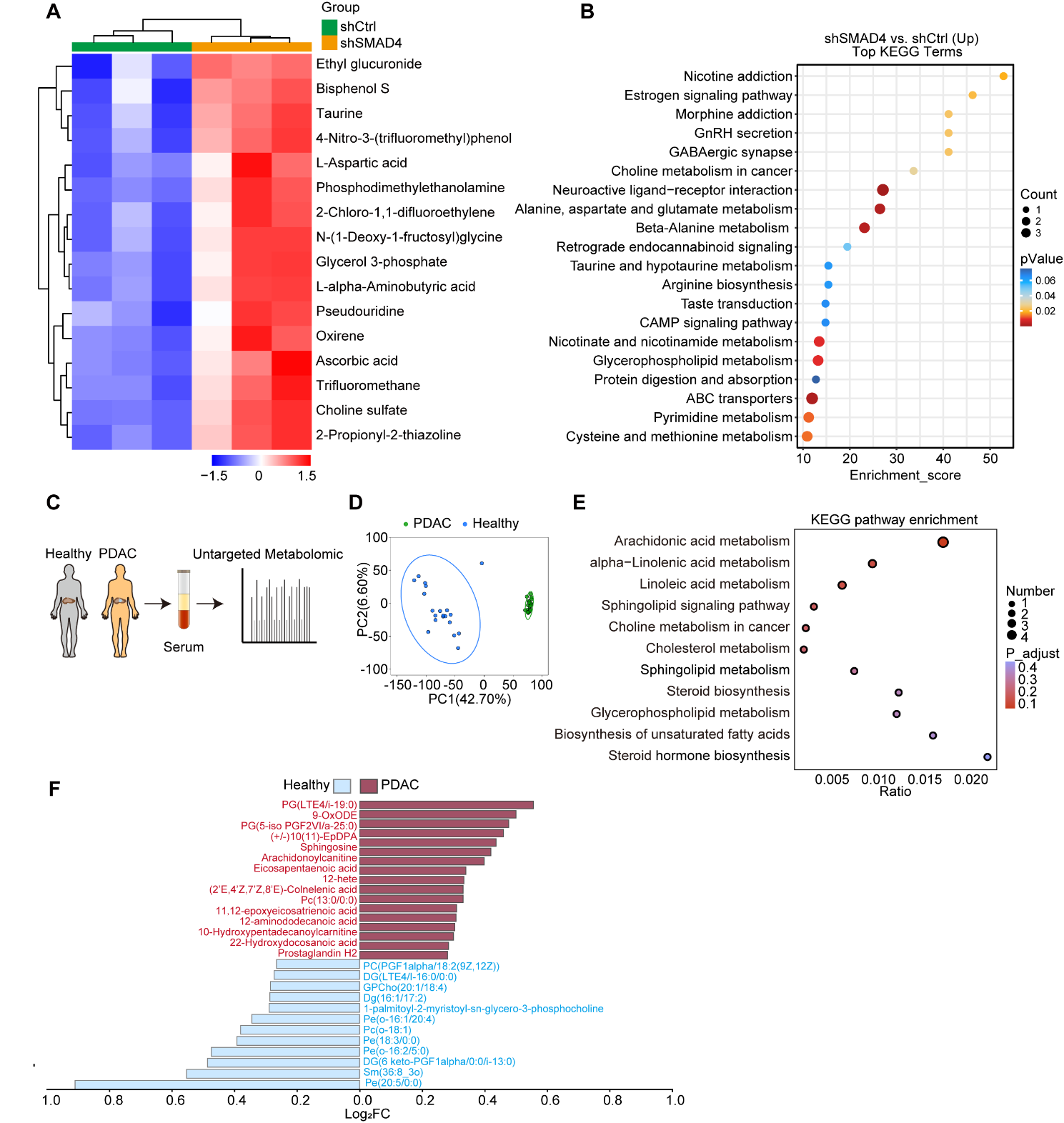


**Figure S1. Untargeted metabolomics reveals metabolic reprogramming in SMAD4-deficient PDAC patient serum.**

**(A)** Heatmap showing lipid metabolites with upregulated abundance in shSMAD4 tumors compared to shCtrl. **(B)** KEGG pathway enrichment analysis of the upregulated metabolites in shSMAD4 cells compared to shCtrl. **(C)** Schematic workflow of the untargeted metabolomics study performed on serum samples from healthy donors and PDAC patients. **(D)** PCA score plot showing the distinct separation of metabolic profiles between healthy controls (blue, n=20) and PDAC patients (green, n=20). **(E)** KEGG pathway enrichment analysis of the differentially abundant metabolites identified in PDAC patient serum. Key lipid metabolism-related pathways, such as sphingolipid and arachidonic acid metabolism, are enriched. **(F)** Bar plot displaying the top significantly dysregulated metabolites in PDAC serum compared to healthy controls. Red bars indicate metabolites upregulated in PDAC, while blue bars indicate those downregulated in PDAC.


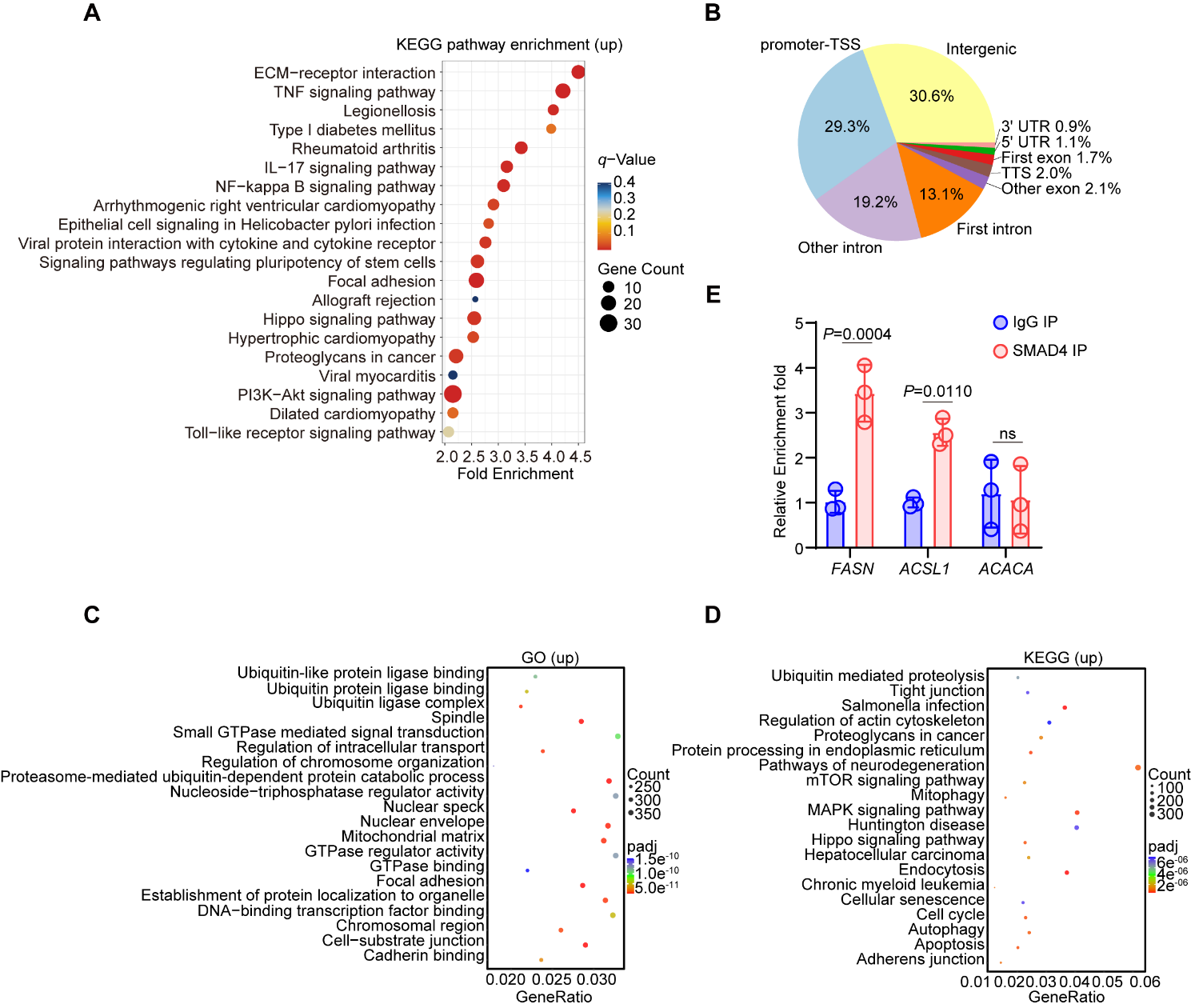


**Figure S2. Genome-wide binding profiles of SMAD4-regulated pathways.**

**(A)** KEGG pathway enrichment analysis of the upregulated genes in shSMAD4 cells. Displaying the top enriched pathways with the smallest q-values. (**B)** Genomic distribution of SMAD4 binding peaks identified by CUT&Tag assays. The pie chart illustrates the proportions of peaks located in promoter-TSS regions, intergenic regions, introns, and exons. (**C**) Gene Ontology (GO) enrichment analysis of the SMAD4 binding regions. (**D**) KEGG pathway enrichment analysis of genes identified as direct transcriptional targets of SMAD4. The analysis was performed on genes associated with significant SMAD4 binding peaks (identified by comparing SMAD4 IP vs. IgG control). **(E)** CUT&RUN-qPCR validation of SMAD4 binding enrichment at the promoter regions of *FASN*, *ACSL1*, and *ACACA*. IgG was used as a negative control. Data are presented as mean ± SD. Statistical analyses were performed by two‐way ANOVA.


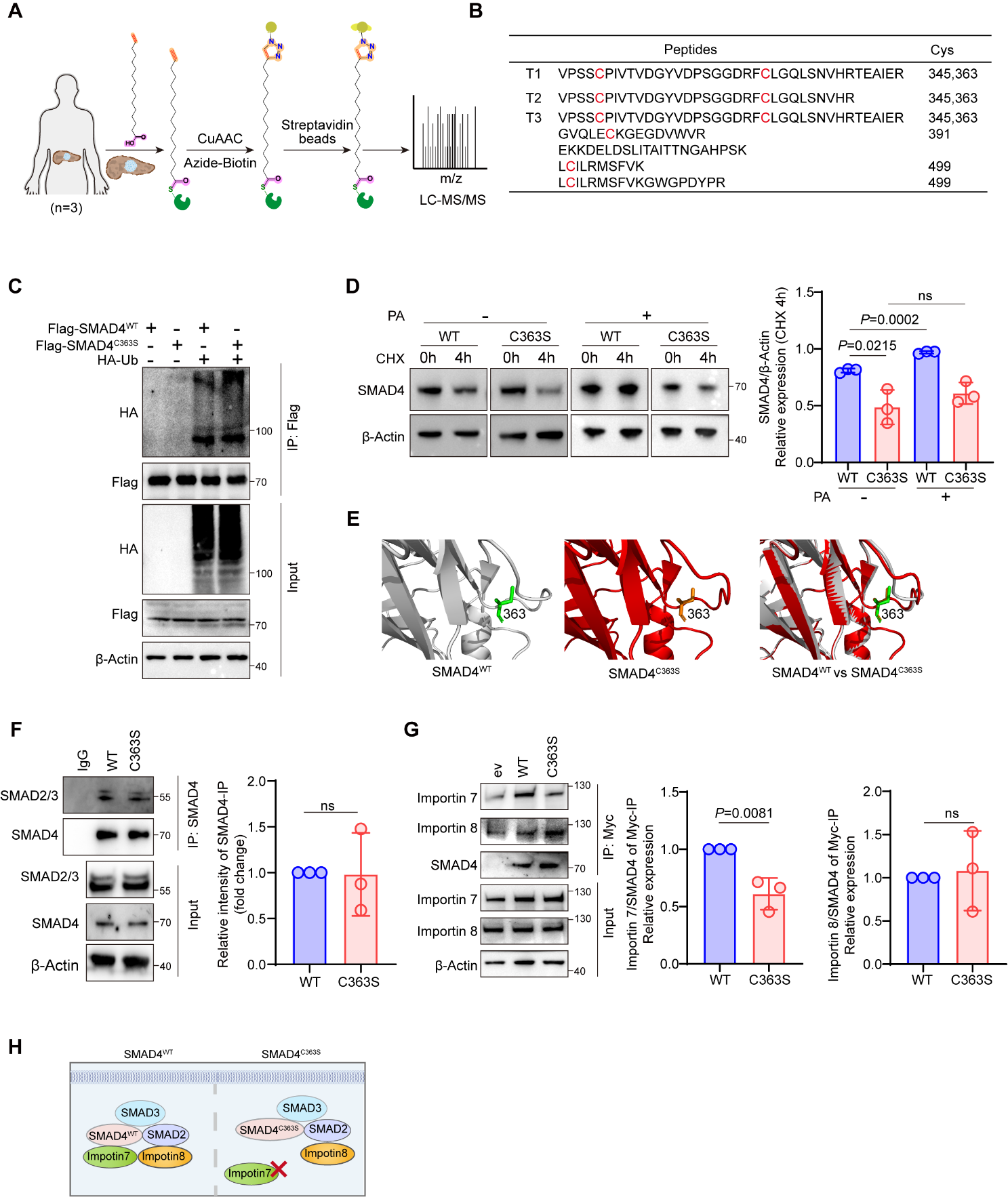


**Figure S3. SMAD4 palmitoylation enhances its protein stability and facilitates interaction with importins.**

**(A)** Schematic workflow for labeling substrate proteins using the click chemistry method to identify palmitoylated proteins in PDAC patient tissues (n=3). **(B)** Peptides of SMAD4 identified in PDAC tissues. Key cysteine residues, including C363 and C499, are highlighted in red. **(C)** Ubiquitination assay. HEK293FT cells were transfected with HA-Ubiquitin and Flag-SMAD4 (WT or C363S). Lysates were immunoprecipitated (IP) with anti-Flag beads and immunoblotted with anti-HA antibody. The C363S mutant exhibited enhanced polyubiquitination levels compared to WT. **(D)** Cycloheximide (CHX) chase assay analyzing the protein stability of SMAD4^WT^ and SMAD4^C363S^ in the presence or absence of exogenous palmitic acid (PA) treatment. The quantification of SMAD4 protein levels in **(D). (E)** Structural modeling showing the local conformation of wild-type (WT) SMAD4 and the C363S mutant. **(F)** Co-IP assay analyzing the interaction between SMAD4 and SMAD2/3. Lysates from cells expressing WT or C363S SMAD4 were immunoprecipitated with anti-SMAD4 antibody and blotted for SMAD2/3. **(G)** Co-IP assay analyzing the interaction between SMAD4 and nuclear transport receptors (Importin 7 and Importin 8). (**H**) Schematic model illustrating the dual mechanism. Left: ZDHHC22 palmitoylates SMAD4, which prevents ubiquitination-mediated degradation and recruits Importin 7. Right: Loss of palmitoylation (C363S) promotes ubiquitination and impairs Importin 7 binding despite intact SMAD2/3 interaction. Data are presented as mean ± SD. Statistical analyses were performed by two‐way ANOVA or Student’s t‐test.


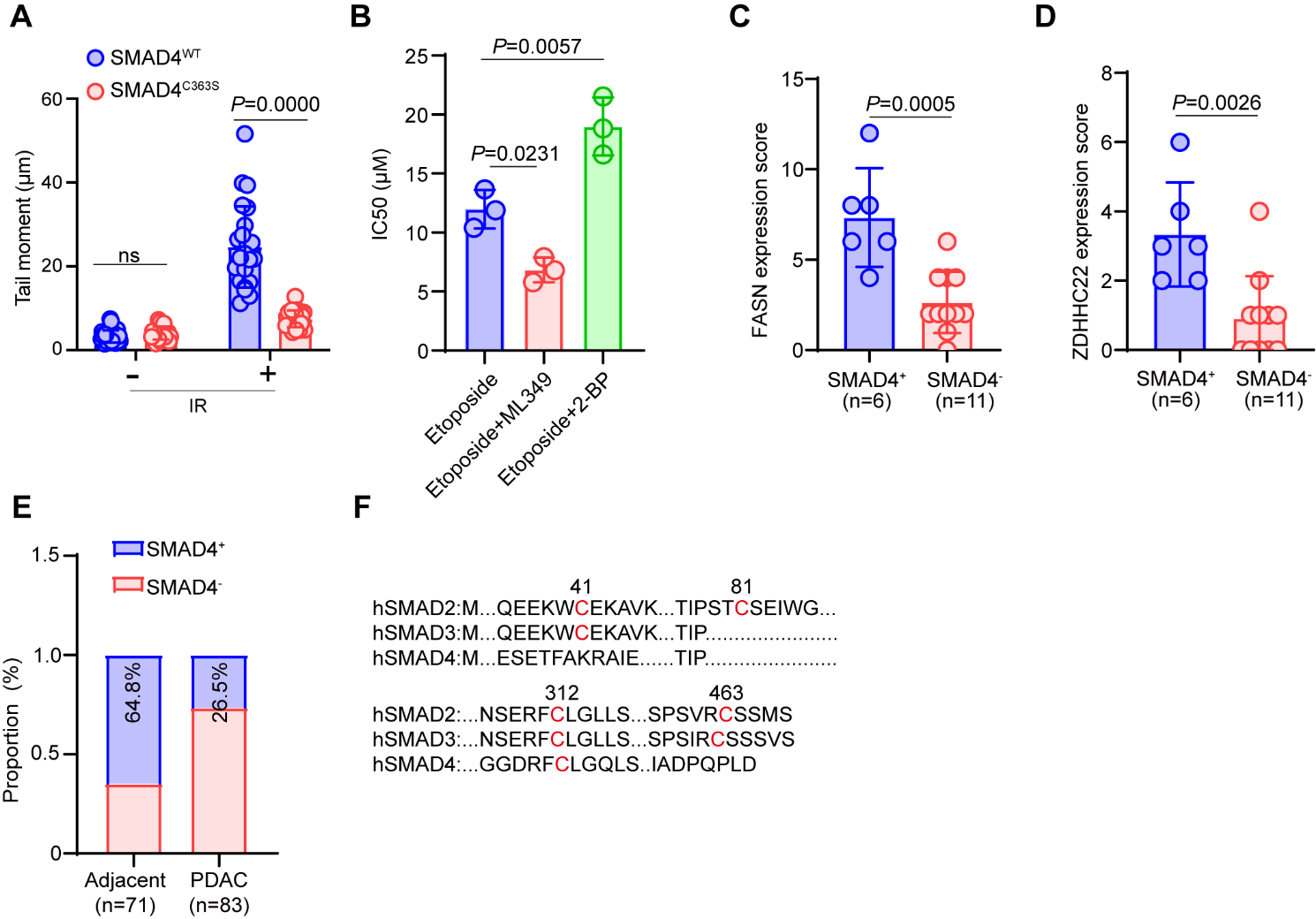


**Figure S4. SMAD4 palmitoylation renders pancreatic cancer cells sensitive to chemoradiotherapy.**

**(A)** Quantification of the tail moment from the comet assay. **(B)** IC50 values of Etoposide in Panc-1 cells co-treated with ML349 or 2-BP. Inhibition of depalmitoylation (ML349) sensitized cells to chemotherapy. **(C)** Quantification of FASN expression scores in the SMAD4^+^ (n=6) and SMAD4^-^ (n=11) groups. SMAD4^+^ tumors displayed significantly higher FASN levels. **(D)** Quantification of ZDHHC22 expression scores in the SMAD4^+^ (n=6) and SMAD4^-^ (n=11) groups. SMAD4^+^ tumors displayed significantly higher FASN levels. **(E)** Statistical analysis of SMAD4 expression in paired adjacent non-tumor tissues (n=71) and PDAC tissues (n=83). **(F)** Sequence alignment of human SMAD2, SMAD3, and SMAD4 proteins. The reported palmitoylated cysteine residue in SMAD2 or SMAD3. Data are presented as mean ± SD. Statistical analyses were performed by Student’s t‐test or one‐way ANOVA or two‐way ANOVA.
